# Colitis in a transgenic mouse model of autoimmune uveitis may be induced by neoantigen presentation in the bowel

**DOI:** 10.1038/s41598-022-27018-9

**Published:** 2023-01-23

**Authors:** C. Mölzer, Y.-H. Liu, E. Muckersie, I. P. Klaska, R. Cornall, H. M. Wilson, L. Kuffová, J. V. Forrester

**Affiliations:** 1 Institute of Medical Sciences, University of Aberdeen, Foresterhill, Aberdeen, AB25 2ZD UK; 2 Nuffield Department of Medicine, Henry Wellcome Building for Molecular Physiology, University of Oxford, Old Road Campus, Headington, Oxford, OX3 7BN UK; 3 Eye Clinic, Aberdeen Royal Infirmary, NHS Grampian, Aberdeen, UK; 4 Present Address: Department of General Surgery, Division of Visceral Surgery, Medical University of Vienna, Vienna General Hospital, Währinger Gürtel 18-20, 1090 Vienna, Austria; 5 Present Address: Flow Facility, University of Glasgow, Wolfson Wohl Cancer Research Centre, Switchback Road, Bearsden, G61 1BD Glasgow UK; 6 Present Address: Centre for Gene Therapy and Regenerative Medicine, Guy’s Hospital, Great Maze Pond, London, SE1 9RT UK

**Keywords:** Immunology, Antigen processing and presentation, Autoimmunity, Inflammation, Innate immune cells, Innate immunity, Mucosal immunology

## Abstract

Undifferentiated uveitis (intraocular inflammation, IOI) is an idiopathic sight-threatening, presumed autoimmune disease, accountable for ~ 10% of all blindness in the developed world. We have investigated the association of uveitis with inflammatory bowel disease (IBD) using a mouse model of spontaneous experimental autoimmune uveoretinitis (EAU). Mice expressing the transgene (Tg) hen egg lysozyme (HEL) in the retina crossed with 3A9 mice expressing a transgenic HEL-specific TCR spontaneously develop uveoretinitis at post-partum day (P)20/21. Double transgenic (dTg TCR/HEL) mice also spontaneously develop clinical signs of colitis at ~ P30 with diarrhoea, bowel shortening, oedema and lamina propria (LP) inflammatory cell infiltration. Single (s)Tg TCR (3A9) mice also show increased histological LP cell infiltration but no bowel shortening and diarrhoea. dTg TCR/HEL mice are profoundly lymphopenic at weaning. In addition, dTg TCR/HEL mice contain myeloid cells which express MHC Class II-HEL peptide complexes (MHCII-HEL), not only in the inflamed retina but also in the colon and have the potential for antigen presentation. In this model the lymphopenia and reduction in the absolute Treg numbers in dTg TCR/HEL mice is sufficient to initiate eye disease. We suggest that cell-associated antigen released from the inflamed eye can activate colonic HEL-specific T cells which, in a microbial micro-environment, not only cause colitis but feedback to amplify IOI.

## Introduction

Undifferentiated uveitis (intraocular inflammation, IOI)^1,2^ is a suspected autoimmune disease (AD) of unknown etiology, accountable for 10% of all cases of blindness in the developed world^3^. In an AD, the immune system is considered to have lost its ability to differentiate between self and foreign antigens, culminating in a pathological immune response against self-antigen. Mechanisms whereby self-antigens become immune targets include post-translational modifications such as citrullination in rheumatoid arthritis (reviewed in ref.^4^). However, in IOI clinical evidence for specific autoantigen reactivity remains limited and we have previously argued that undifferentiated IOI may be pathogenetically driven by infectious agents^5,6^. Experimentally, however, there are numerous examples of IOI induced by specific ocular antigens and their peptides, particularly the photoreceptor-specific antigen interphotoreceptor retinol binding protein (IRBP)-induced experimental autoimmune uveoretinitis (EAU) model^7,8^. EAU is a CD4+ Th17, Th1 T cell-mediated disease. More recently, EAU has been shown to require an intact, homeostatic microbiome, both in adjuvant-based IRBP-peptide-inducible EAU and in spontaneously developing EAU in an IRBP T cell receptor (TCR) transgenic mouse^9–14^. How the microbiome contributes to this T cell mediated autoimmune disease is not clear, but several mechanisms have been proposed, including bystander activation and cross-reactivity between commensal antigen and IRBP^15^. In contrast, in an ovalbumin (OVA)-induced model of colitis, evidence for dual TCR specificity has been shown whereby OVA-specific T cells also respond to colonic antigen^16^ although evidence for a similar mechanism was not found in the spontaneous EAU model^15^.

We have investigated these mechanisms using a transgenic mouse model in which hen egg lysozyme (HEL) is expressed in the retinal photoreceptor layer (single transgenic, sTg IRBP:HEL mice; referred to here as “sTg HEL”). When crossed to the sTg 3A9 TCR mouse (referred to as “sTg TCR”), double transgenic (dTg 3A9 TCR IRBP:HEL mice, referred to hereafter as “dTg” mice) develop spontaneous EAU (at post-partum day P20-21) with 100% penetrance^17^ despite profound thymic depletion-linked lymphopenia. Here we show that dTg mice also develop clinical and pathological granulomatous colitis (~ P30) in which there is extensive infiltration of the colon with myeloid cells and HEL antigen-specific T cells. Using a monoclonal antibody (C4H3) which recognizes an MHC Class II-HEL peptide [46–61] complex (MHCII-HEL) we provide evidence of potential HEL-antigen presentation in both the retina and the colon.

We propose that EAU-inducing HEL-specific T cell expansion is initially generated by lymphopenia in this model (~ P21) and the subsequent retinal damage permits cell-associated extraocular antigen to traffic to the gut where activation of HEL-specific T cells, in a microbial-rich microenvironment, induces colitis and amplifies ocular inflammation.

## Results

### Double-transgenic (dTg) mice with spontaneous uveitis also develop colitis

**Figure 1:**
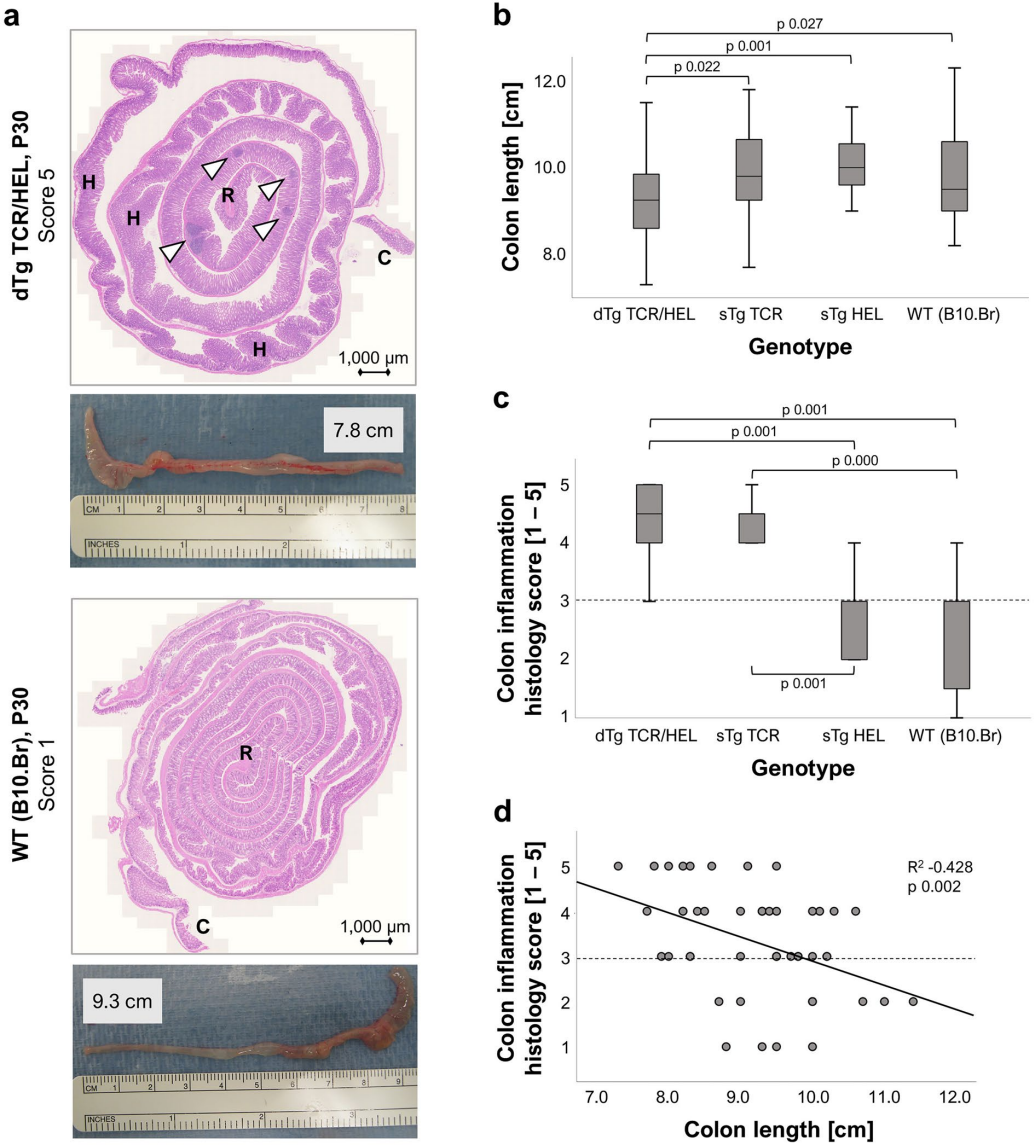
Mouse large intestines exhibit genotype-specific histological features. (**a**) H&E-stained colon section (Swiss roll) of dTg mice shows clear signs of inflammation (granulomatous colitis). Cardinal features are granulomas/crypt abscesses (white arrowheads) and crypt hyperplasia (H), along with bowel shortening and oedema. WT mice have longer bowels with normal appearance of mucosal epithelium. Abbreviations: R, rectum; C, caecum. (**b**) Colons of dTg mice are significantly shorter than those of all other genotypes; (**c**) Both dTg and sTg TCR colons have significantly more inflamed phenotypes as compared to the sTg HEL and WT mice. (**d**) Colon length and histological inflammation score are significantly correlated. Dashed lines indicate clinically irrelevant “background” colon inflammation. Non-parametric statistical procedures were used; significance scored at *p* ≤ 0.05. For each genotype group n = 10–13 colons were independently examined. Mice were aged P30 ± 3 days.

Double-transgenic mice develop HEL-specific autoimmune uveitis at weaning (P20/21) and reach peak inflammation (Grade 3–4 EAU) by P30^17^. These findings were confirmed in this study. dTg mice also spontaneously developed clinical signs of colitis at ~ P28/30 (diarrhoea, + /− rectal prolapse and bleeding). We therefore investigated dTg mice with clinical colitis and compared them to sTg HEL, sTg TCR and WT mice. Of the four genotypes, only dTg developed clinical signs of colitis. In addition, only dTg mice showed gross pathological signs of colitis (bowel shortening) (Fig. 1). For histological analysis, colons were prepared using the "Swiss roll" technique allowing analysis of the entire colon. Histologically, but not clinically sTg TCR mice had signs of increased inflammatory cell infiltration in the colon compared to sTg HEL and WT mice but no bowel shortening and no clinical signs. sTg HEL mice showed minimal evidence of inflammatory cell infiltration and histology was similar to non-transgenic WT mice (Fig. 1; Suppl. Fig. S3).

Histological (H&E) signs of colitis in this model included granulomas, crypt abscesses and "erupting" Peyer’s patches where inflammatory cells appeared to have penetrated the submucosal *muscularis* layer and invaded the lamina propria (LP). These signs were seen in both dTg and sTg TCR mice (Fig. 1 and Suppl. Fig. S3). They were almost absent in sTg HEL and WT mice (Suppl. Fig. S4) which only exhibited mild (“baseline”) inflammation (Fig. 1). In particular, “control” colons of sTg HEL and WT mice did not show the structural breakdown ("eruption") in Peyer's patch morphology, but instead retained a normal appearance (Suppl. Fig. S4 and Suppl. Table S3).

These findings were semi-quantitatively assessed. Colon inflammation was scored following a published scoring system for induced colitis^18^, with modifications (Suppl. Table S2) to take into account the spontaneous nature of colitis in the present model and the histological differences, particularly with regard to tissue swelling observed in classical experimental models^19^. As can be seen from Fig. 1b, colon shortening of dTg mice was statistically significant compared to all other genotypes. Changes in colon length negatively correlated with histological signs of colitis as previously reported in other models^20,21^ (Fig. 1d). However, while colons of dTg and sTg TCR mice shared comparable histological inflammatory scores (4–5, marked to severe), only dTg mice had signs of bowel shortening and thickening/swelling. This was accompanied by the clinical signs of diarrhoea, and in severe cases, rectal prolapse and bleeding.

**Figure 2:**
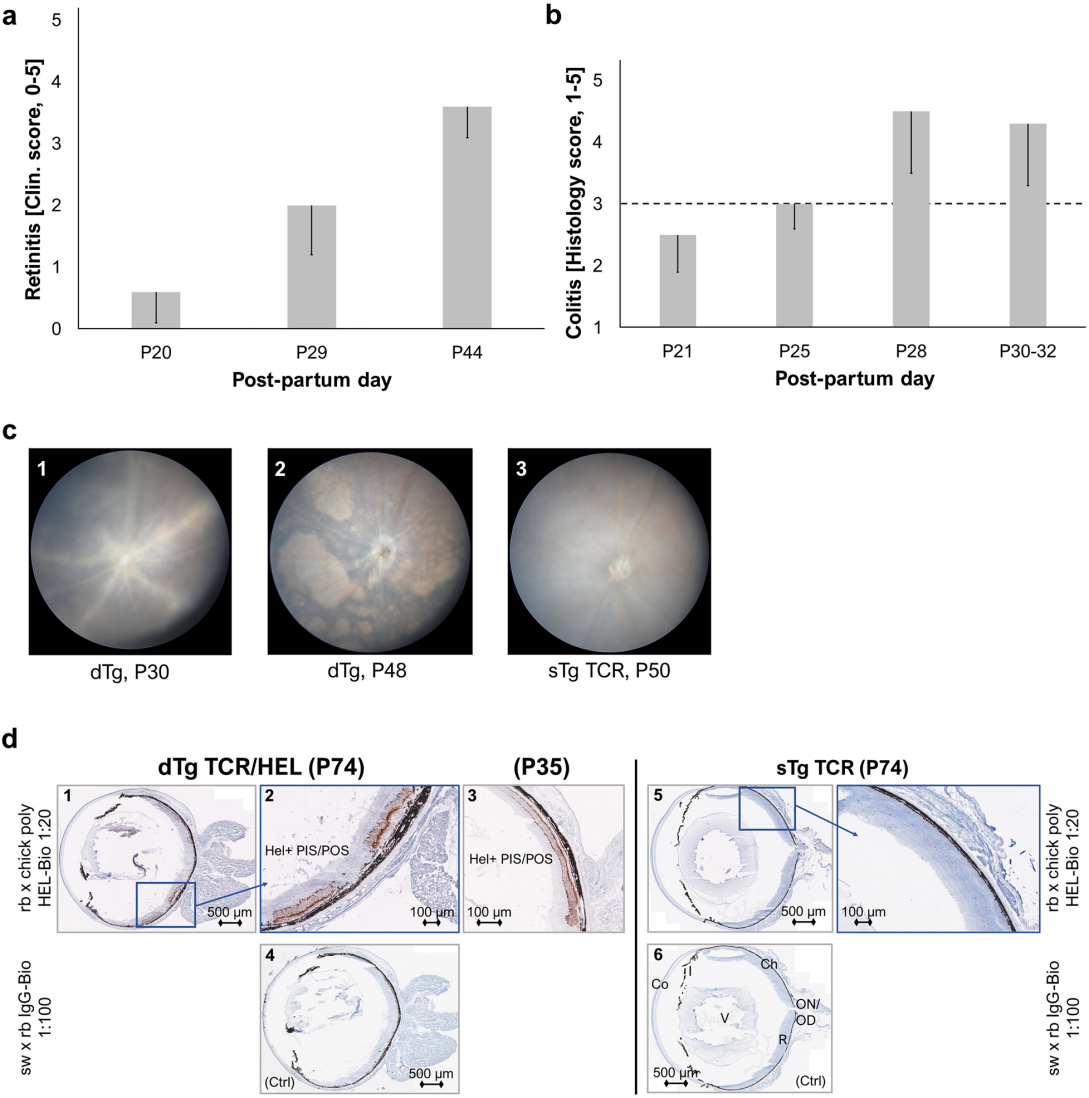
Colitis and retinitis (spontaneous EAU) have distinct clinical onsets. (**a**) Mice with EAU (n = 7–8, assessed independently) spontaneously develop uveitis from P20 with gradual worsening of inflammation until P44 when the disease wears off leaving behind irreversible retinal atrophy. (**b**) Clinical signs of colitis (i.e., diarrhoea, rectal prolapse) were observed in dTg mice (n = 4 per age-group, independently assessed) from around P30. Histological staining and subsequent scoring revealed the presence of colon inflammation after P25 (clinical scores between 1 and 3 were identified as the baseline range, dashed line). Error bars indicate sd. (**c**) Representative fundoscopy images of P30 dTg (1, vasculitis), P48 dTg (2, retinal atrophy), and P50 TCR eyes (3, normal retina) (n = 34 eye pairs/genotype group; independently assessed. (**d**) Eye sections of dTg aged mice (P74; n = 3, independently stained, three replicates) show transgenic expression of HEL protein in their photoreceptor layer (1, 2; brown staining). This is specific to this spontaneous EAU mouse model in which HEL is the uveitogenic antigen^17^. Photoreceptor structure is better preserved in younger eyes (3). Note the absence of staining in control section (ctrl; 4) as well as sTg TCR eyes that are devoid of the retinal transgene (5, 6). Abbreviations: PIS/POS, photoreceptor layer inner and outer segment; Ch, choroid; R, retina; ON/OD, optic nerve/disc; V, vitreous; Co, cornea.

We further explored the time of onset of EAU (Fig. 2a) in relation to colitis onset (Fig. 2b). As indicated above, inflammatory signs of EAU in dTg mice present at P20/21^17^, become maximal at approximately P30-44 and decline thereafter (Fig. 2c). This retinal decline is demonstrated by staining retinal sections for photoreceptor HEL expression (Fig. 2d). Clinical signs of colitis (diarrhoea, rectal prolapse) and macroscopic bowel shortening in dTg mice develop at P28/30 when EAU is approximating its peak. Microscopic signs of colitis in dTg mice develop after P25 (Fig. 2b). However, while at P21 IOI was clinically present, there were no histological or clinical signs of bowel inflammation in dTg mice.

### Double-transgenic (dTg) mice with colitis have increased numbers of LP Treg

**Figure 3:**
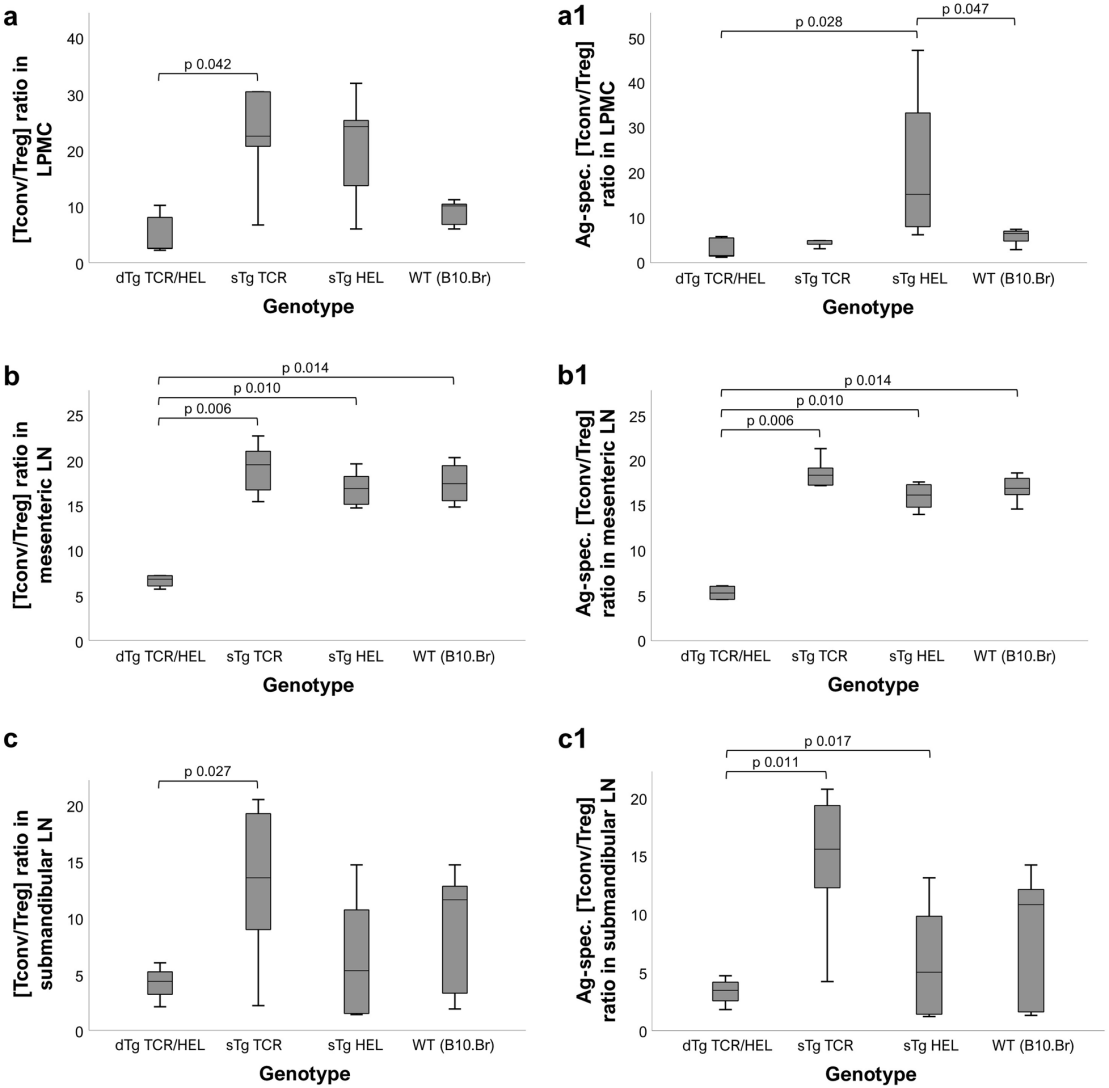
Mice with the dTg genotype have relatively lower (antigen-specific) [Tconv/Treg] cell ratios across different tissues. (**a**, **a1**) in LPMC (lamina propria mononucleated cells); (**b**, **b1**) in mesenteric lymph nodes (LN); and (**c**, **c1**) in submandibular (eye-draining) LN. dTg mice were found to have significantly lower [Tconv/Treg] quotients across all tissues as compared to their non-lymphopenic counterparts. The same was true with reference to antigen-specificity. Treg cells had been defined as CD4+ CD25+ FoxP3+, Tconv as CD4+ CD25± FoxP3-. Antigen-specific populations had been classified as Vbeta8.1/8.2+ ^17^. Ratios were calculated based on absolute cell numbers [n] acquired using flow cytometry. Non-parametric statistical procedures were used, significance taken as *p* ≤ 0.05. Per genotype group n = 5–7 mice (aged P30 ± 3) were used across 2–3 independent experiments.

We next investigated the T cell populations in the colon compared to the eye. Specifically, we looked at T cell populations in the LP and mesenteric lymph nodes (mLN) draining the colon. dTg mice develop EAU despite profound CD4 lymphopenia^17^ which progressively lessens as the mice age although they are still relatively lymphopenic at P30^22^. Lymphopenia in dTg mice includes a reduction in the absolute numbers of Treg in EAU mice and EAU can be prevented by adoptive transfer of antigen-specific Treg^22^. dTg mice with colitis also have profound lymphopenia affecting the LP T cell population but have proportionately more Treg in the colon than Tconv cells compared to all other genotypes (Fig. 3a, Suppl. Tables S4 and S5). They also have three times the absolute numbers of LP Treg than sTg TCR mice despite having 60% fewer Tconv cells (Suppl. Table S4). However, the relative proportion of LP antigen-specific [Tconv/Treg] T cells was considerably less in dTg mice compared to sTg TCR mice (Fig. 3a1) while the absolute number of LP Tconv was similar in dTg and sTg TCR mice despite the systemic lymphopenia in dTg mice (Suppl. Tables S4 and S5). Interestingly, these differentials in relative proportions in [Tconv/Treg] ratios extended to the eye draining (submandibular) and colon draining (mesenteric) lymph nodes in dTg mice vs sTg TCR mice (Fig. 3b, b1 and Fig. 3c, c1).

### MHCII-HEL antigen complexes are detectable on granulocytes with specific antigen presenting properties in the eye and colon of dTg mice

**Figure 4:**
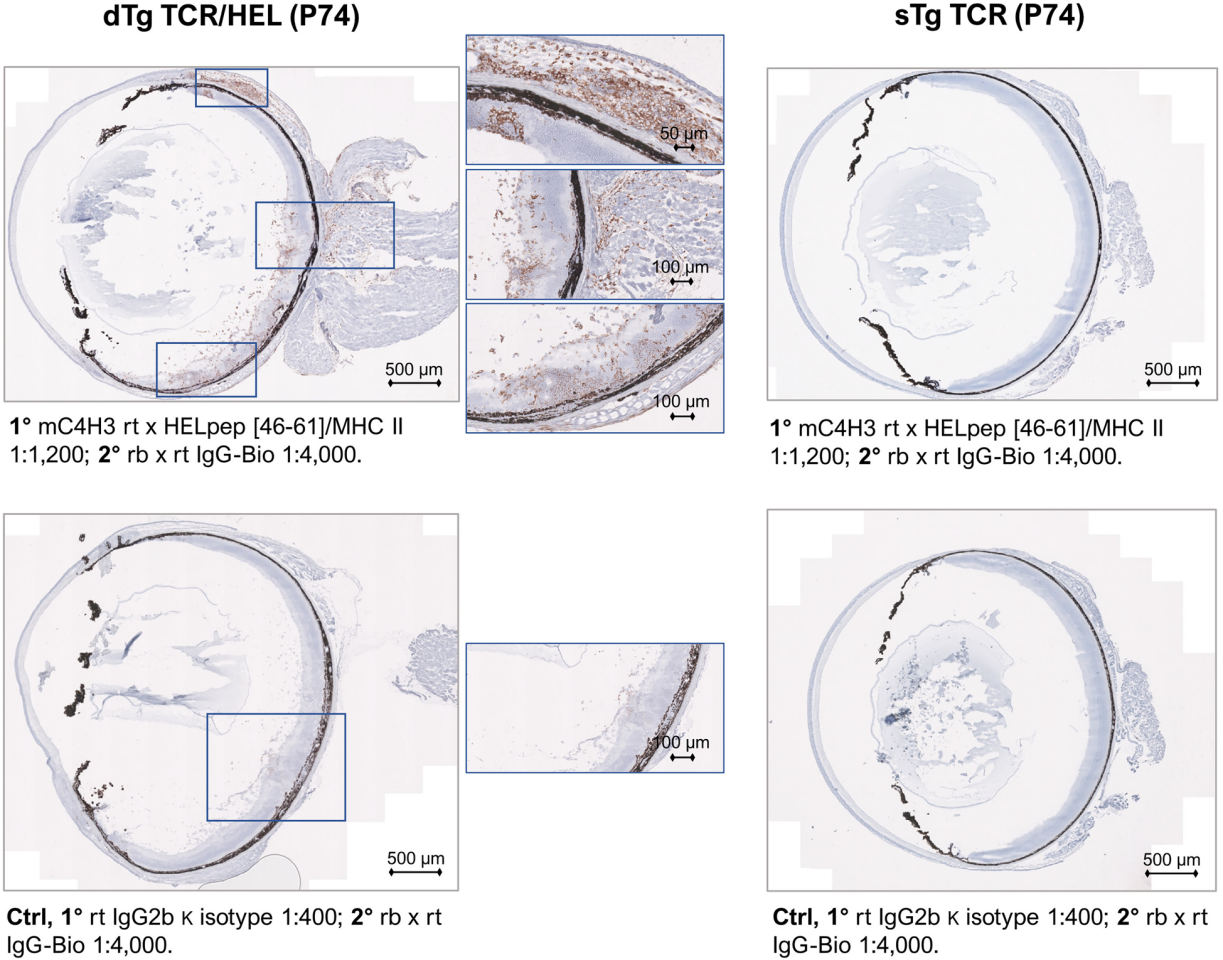
MHCII-HEL complexes are exclusively present in retinas of dTg mice. The monoclonal C4H3 rat anti-chicken antibody towards HEL peptide [46–61] was used to stain MHCII-HEL complexes in eye sections. OCT sections were counterstained with Haematoxylin QS (Vector Laboratories, 2BScientific, UK). Left panel: P74 section from dTg mouse; note positive brown staining particularly in and around inflamed retinal tissue and uveal tract vs. control (faint background only). Right panel: staining was performed using sTg TCR eye sections. No staining was found in either sTg TCR test conditions. Per genotype group n = 3 sections were independently stained across three replicates.

**Figure 5:**
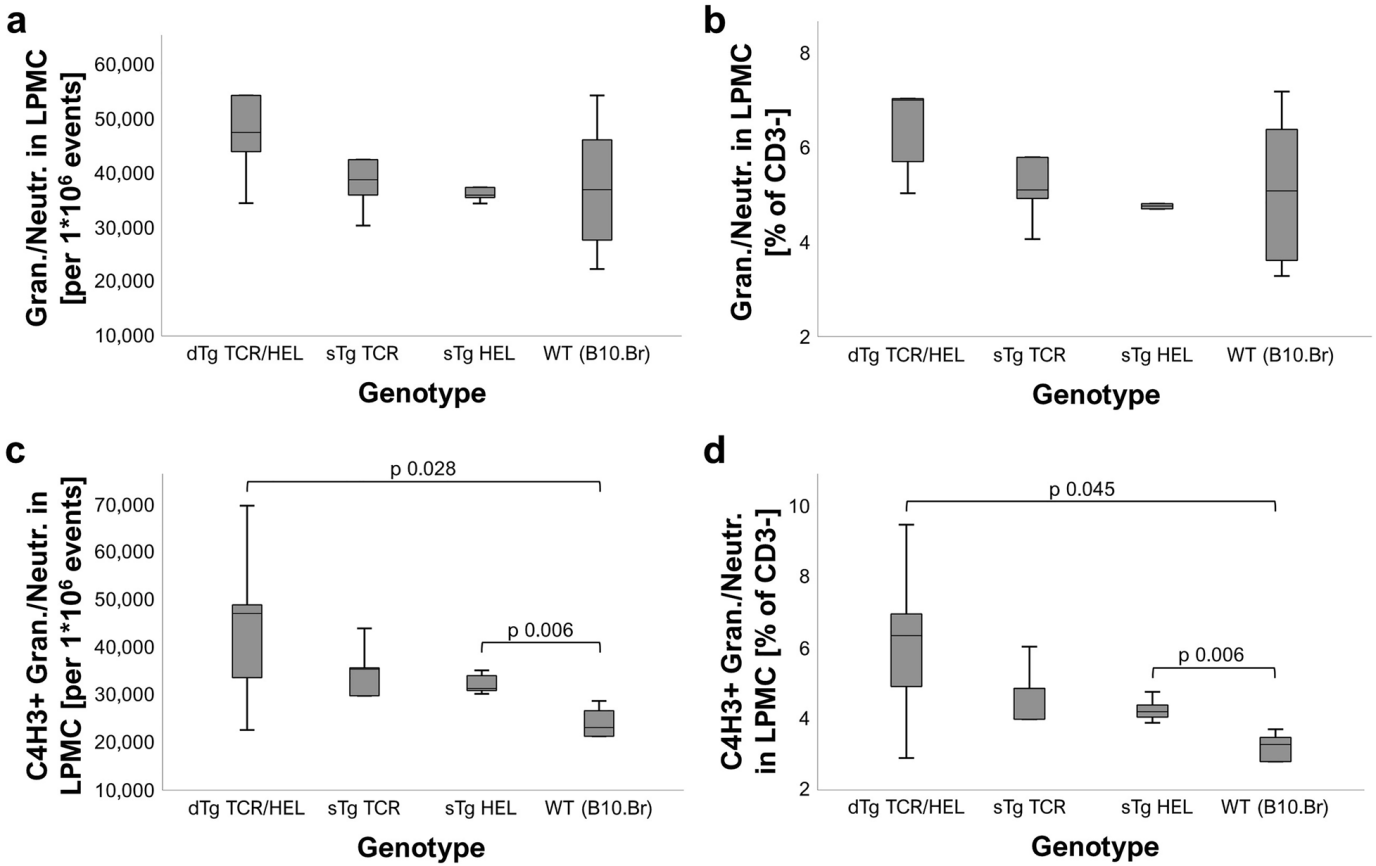
MHCII-HEL staining positivity is significantly stronger in lamina propria mononucleated cells isolated from dTg colon. MHCII-HEL complexes were detected using flow cytometry in granulocytes/neutrophils in LPMC (lamina propria mononucleated cells) isolated from mouse colon (aged P30 ± 3). While no difference was found in either (**a**) total granulocyte/neutrophil numbers recorded, and (**b**) cell counts relative to all CD3- events, there were significantly more MHCII-HEL presenting cells [C4H3+; (**c**) and (**d**)] found in LPMC isolated from dTg mice as compared to all other genotypes (= background level staining). This result is indicative of potential for HEL antigen presentation capacity in colons of dTg mice. Cells had been defined as CD3- CD11b+ Gr1/Ly6+. Non-parametric statistical procedures were used, significance taken as *p* ≤ 0.05. Per genotype group n = 5–7 mice were used across 2–3 independent experiments.

**Figure 6:**
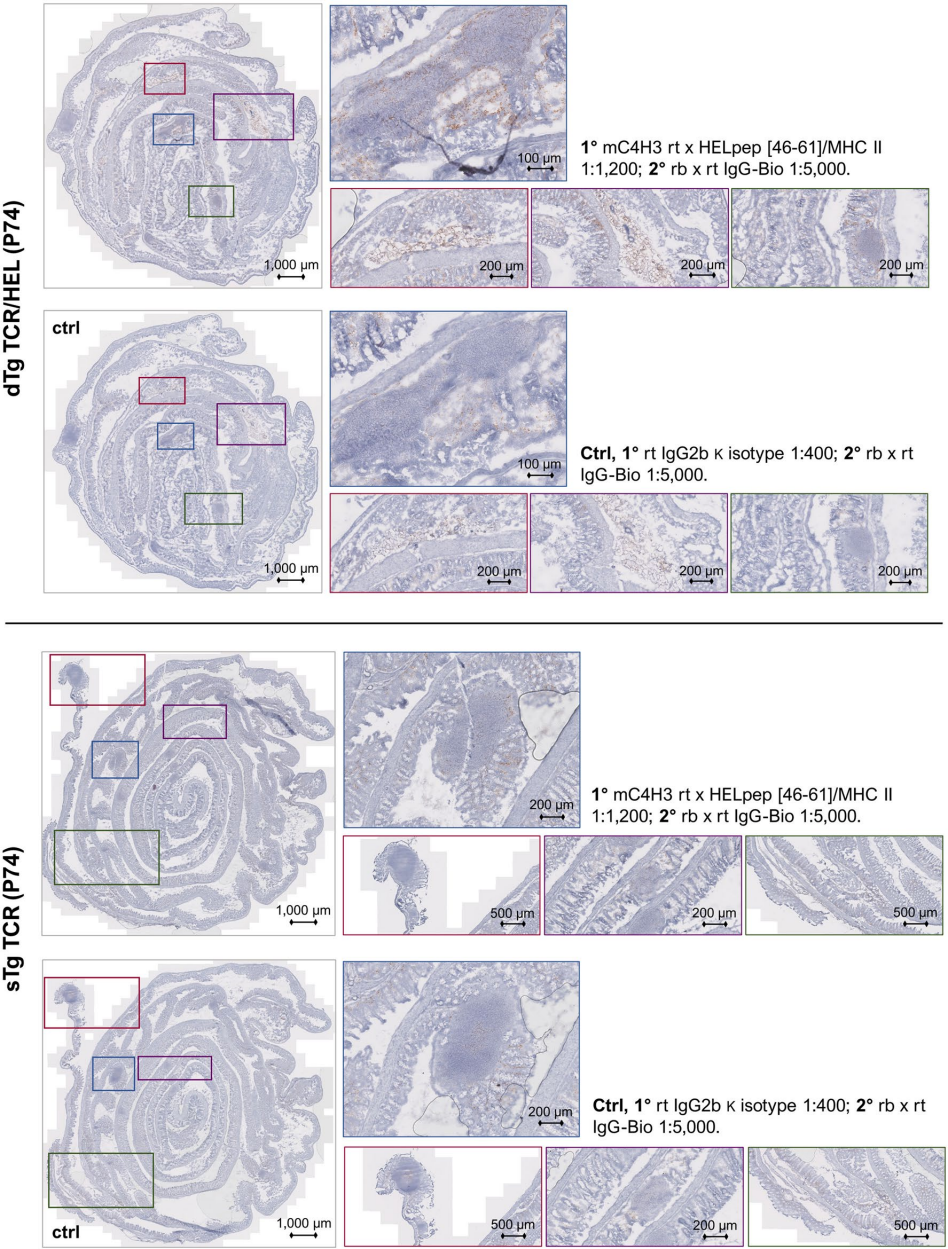
MHCII-HEL complexes are exclusively present in colon of dTg mice. The monoclonal C4H3 rat anti-chicken antibody towards HEL peptide [46–61] was used to histologically stain MHCII-HEL complexes in colon Swiss roll sections. Sections were counterstained with Haematoxylin QS (Vector Laboratories, 2BScientific, UK). Top panel: P74 section from dTg mouse; note positive brown staining particularly in and around granulomas and erupting Peyer’s patches vs. control. Bottom panel: staining was performed using sTg TCR colon sections. Only faint background staining was found in either sTg TCR test conditions. Note: Unavoidable faint background staining results from the intrinsic abundance of inflammatory cells in the lamina propria. Per genotype group n = 3 sections were independently stained across three replicates.

The above data concerning HEL-specific Tconv and Treg suggested the possibility that colitis might be driven by activation of T cells locally in the colon as well as in the eye. We therefore explored the possibility that HEL neoantigen presentation might occur in the colon, using a monoclonal antibody (mC4H3) directed against the HEL-peptide [46–61] MHC class II antigen complex^23,24^, both by flow cytometry (in the CD3-, MHC Class II+, CD11b+, Gr1+ populations) and by immunohistochemistry (Suppl. Figs. S5 and S6). In the retina, C4H3 + (MHCII-HEL +) cells were identified within granulomatous lesions and on individual cells throughout the inflamed retina and uveal tract (Fig. 4). In the colon LP, MHCII-HEL complexes on antigen presenting cells were detected both by flow cytometry (Fig. 5) and immunohistochemistry (Fig. 6) in dTg mice but was significant only in the granulocyte (Gr1 +) population. Interestingly, sTg HEL mice also showed significant levels of C4H3 + granulocytes (Fig. 5) suggesting that cell-associated retinal HEL antigen trafficking occurs constitutively from sTg HEL mice as has been reported using RT-PCR^25^. These data imply a potential for antigen presentation of HEL to (antigen-specific) T cells via granulocytes in dTg mice which have been shown in previous studies to selectively activate resident memory T cells.

### Correlations between EAU, colitis and the T cell transgene

In view of the signs of LP inflammatory cell infiltration in sTg TCR and dTg mice we wished to identify any potential role for the presence of the transgenic TCR and clonal dominance in the T cell repertoire on LP T cell populations and the risk of colitis. Non-parametric bivariate linear correlation (Spearman *rho*) was completed across the entire model (i.e., including all genotypes) to determine statistical connection across variables relating to the colitis as determined by clinically overt disease (colon length) and histological signs.

Subsequently, all respective biologically meaningful, correlated independent variables were included in a linear stepwise regression analysis divided by the two genotype groups (i.e., dTg and sTg TCR, respectively). The clinical endpoint parameters of histological colitis (inflammatory cell infiltration), colon length and retinitis had been defined as the dependent variables. After obtaining initial regression models for each parameter, relevant confounding variables were removed in a stepwise fashion (see table in Suppl. Fig. S7).

In dTg mice, the model suggested a causative relation between antigen-specific Treg cells in retina (adj. R^2^ 0.342, *p* 0.001) and observed statistical variation in microscopic (histological) colitis. The same was true for the observed variation in colon length (adj. R^2^ 0.269, *p* 0.021). This result emphasises the strong bivariate relationship (correlation) determined between inflammatory cell infiltration (microscopic colitis) and colon length (R^2^ −0.428, *p* 0.002 for all genotypes). These data demonstrate that there is a strong link between inflammation and bowel shortening but inflammatory cell infiltration may occur without inducing bowel shortening and probably reflects the nature and/or activation status of the cells. While sTg TCR mice have normal colon length, they also exhibit marked signs of cell infiltration histologically (microscopic colitis, inflammatory cell infiltration). The observed variation in colitis in sTg TCR mice might be explained by Tconv in retina (presumably circulating/trafficking intravascular Tconv cells). Tconv cell numbers in sTg TCR are minimal since this genotype does not develop retinitis or have retinal tissue-resident CD4+ T cells (Suppl. Fig. S6)^17,22^. Finally, regression analysis proposed that variation in retinitis exclusively occurring in the dTg genotype might have been causatively related to Treg cells in smLN (adj. R^2^ 0.367, *p* 0.001) (Suppl. Fig. S7). The regression analysis supports a potential causative link between pathogenesis of the retinitis and colitis as well as the previously identified link between Treg and protection from retinitis^22^.

## Discussion

The data reported here show that dTg lymphopenic mice, which express the HEL-specific TCR and develop spontaneous uveitis (IOI), also develop severe colitis clinically (diarrheoa, rectal prolapse), macroscopically (colon shortening) and microscopically (LP inflammatory cell infiltration). Lymphopenic mice are prone to colitis when exposed to a microbial microenvironment^26^, particularly helicobacter^27^ which may partly account for the colitis in dTg mice in the present study. WT mice and sTg HEL mice did not develop colitis. In contrast, sTg TCR mice developed LP inflammatory cell infiltration (microscopic colitis) although of lesser severity than dTg and without bowel shortening. As argued below, the role of antigen presentation of HEL peptide to HEL specific CD4+ T cells together with microbial-induced non-specific inflammation in the development of colitis, is supported by the findings (Fig. 5c,d; Suppl. Table S6) that (a) the potential for antigen presentation is significantly higher in dTg vs WT mice but not between sTg TCR and WT mice; and (b) in the absence of colitis, MHCII-HEL+ complexes were significantly more abundant in sTg HEL mice than WT mice, indicating possible ectopic expression of cell-associated retinal HEL protein in this model, and consistent with constitutive trafficking of antigen from the retina as has been shown previously^25^.

An important question here is whether there is a pathogenetic link between the IOI and the colitis. The association between IOI/uveitis and inflammatory bowel disease (IBD) is long recognised both clinically and experimentally^12,28–30^. This association is part of the now well-established link between the gut microbiome and the pathophysiology of diseases generally^31^. However, precisely how gut dysbiosis and IBD are associated with IOI is unclear.

Previous work suggests that IOI may be a risk factor for gut dysbiosis and IBD (colitis)^32^ although experimental studies suggest the reverse may be true^11,15,33^. Specifically, in an IRBP-TCR Tg model of spontaneous autoimmune uveitis (EAU), in which there are ~ 30% IRBP-TCR specific CD4 T cells, it has been proposed that IOI is caused by antigen-specific T cells activated in the bowel by IRBP/commensal-antigen cross-reactive peptides. The possibility that IRBP-specific T cells could be activated by ectopic expression of specific antigen, rather than commensal antigen in the gut was explored in these studies but no evidence for gut expression of IRBP was reported (reviewed in ref.^14,33^). At this time, the identity of the cross-reactive commensal antigen is unknown.

Other work has shown that the gut microbiome is required for the pathogenesis of uveitis induced by inoculation of IRBP peptide in adjuvant^9,12^. A role for the microbiome, possibly via enteric antigen presentation, in IOI is therefore proposed both experimentally and clinically, but the mechanism remains unclear. Enteric antigen presentation has been implicated in the development of spontaneous colitis^16,34^ but not in any associated organ-specific diseases. Ectopic expression of CNS antigens is recognised^35^ while CNS antigen cross reactivity with *Clostridium difficile* is suggested in MS^36^. Thus, there is circumstantial evidence for cross-reactivity between commensal and enteric antigens and CNS antigens.

Granulomatous inflammation is a major feature of T cell driven colitis^20,37^. In the acute TNBS-induced colitis model, IFNγ-producing Th1 cells dominate the pathology, while Th17 cells are also involved and may have both an effector and a regulatory role (reviewed in ref.^20^). In dTg mice both Th1 and Th17 cells cause the retinal pathology^22^ and are likely to be involved in the colitis. Indeed, the granuloma formations in the colon reported here as one of the cardinal features of spontaneous colitis resembled the tertiary lymphoid follicles, which are a major feature of the retinitis in dTg mice as well as in IRBP Tg mice (Suppl. Figs. S3 and S5)^22^. In some places, the granulomas had progressed to microabscesses with a heavy infiltrate of granulocytes (pyogranulomas) (Fig. 1a, Suppl. Figs. S3 and S5). In addition to the granulomas and microabscesses, there was widespread inflammatory cell infiltration throughout the LP, in some instances with invasion of the submucosal *muscularis* "capsule" into the LP. There was also a reduction (*p *0.008) in the number of Peyer's patches in dTg and sTg TCR mice compared to sTg HEL and WT mice (Suppl. Table S3).

We surmised that the pathogenesis of colitis in dTg mice reflected mechanisms similar to those which induced retinitis. We have shown previously that despite severe lymphopenia in dTg mice, spontaneous EAU becomes progressively more severe with expansion of HEL-specific Teff cells due to a reduction in the absolute number of peripheral Treg causing an imbalance in the proportion of Teff vs Treg cells^22^. Adoptive transfer of in vitro HEL-activated, but not non-specifically (CD3/CD28) activated, sTg TCR Teff cells into sTg HEL mice also induced EAU. In contrast, adoptive transfer of antigen-experienced Treg completely prevented EAU in this model in dTg mice. Treg are known to control colitis. In early experiments, it was shown that naive CD4+ CD45RB^hi^ cells, lacking CD45RB^lo^ Treg, when adoptively transferred to lymphopenic mice, induce colitis^38–41^ while adoptive transfer of CD45RB^lo^ Treg prevented colitis^42^. Indeed colonic Treg are essential for bowel homeostasis (reviewed in ref.^43^) and Treg IL10 and TGF beta are critical mediators of this effect, as is CTLA4 ligation (reviewed in ref.^44^).

The question which arises therefore is whether an absolute reduction in Treg in the lymphopenic dTg mice, as described here, is alone sufficient to induce colitis as it does retinitis. This is a more complex question in relation to colitis than retinitis since the colon has a constitutive population of immune cells including CD4 Teff and Treg (up to 30% of the total colonic CD4 T cell population in SPF-housed mice are Treg^45^). These cells reside mostly in the LP and increase in number progressively from weaning to adulthood^45,46^. In the current study, the number of CD4+ CD25± Tconv (“Teff”) cells in the LP of dTg mice was around half that of normal WT mice (Suppl. Table S4). In contrast, the number of LP CD4+ CD25+ FoxP3+ Treg in the dTg mice was proportionately greater with a statistically significant difference in the [Tconv/Treg] ratio (Suppl. Tables S4 and S5). Indeed, despite the lymphopenia, the absolute number of LP Treg in dTg mice was greater than in sTg TCR mice. Interestingly this applied only to non-antigen specific Treg and not to HEL-specific Treg. Thus, while lymphopenia in dTg mice affected the total CD4 T cell population in the colon, this did not extend to Treg. Furthermore, the proportional increase in Treg in the colon was replicated in the submandibular and mesenteric lymph nodes in dTg mice compared to the remaining genotypes where the percentage of Treg was closer to predicted levels (~ 5–20%) (Suppl. Table S4).

Overall, these data suggest that in dTg mice in which there was structurally damaging colitis (bowel shortening) with overt clinical signs, LP Treg were comparably less effective in controlling inflammation than in sTg TCR mice, which, as indicated above, had microscopic signs of bowel inflammation (microscopic colitis) but not colon shortening or clinical signs. It is interesting to speculate why LP Treg in dTg mice might be less effective in protecting against colitis. This may have been due to the relative lack of antigen-specific/antigen-experienced Treg rather than a lack of Treg overall since non-antigen-experienced Treg are ineffective in preventing retinitis^22^.

A further question is how and where the colitogenic T cells become activated in this model of EAU. In lymphopenic Tg pigeon cytochrome-c TCR mice, colitis developed when antigen-specific T cells were activated locally in the LP, but this appeared to occur via cross-reaction with enteric antigen^47^. In the same study, there was evidence for endogenous TCR recombination events underpinning some colonic T cell activation. Further studies have shown that both specific commensal antigen activation and homeostatic expansion of T cells are required to induce disease in lymphopenic models of colitis ^13^. In the pathogenesis of retinitis, Caspi's group suggests that cross-reactive commensal antigen activates IRBP-TCR transgenic T cells in the gut, permitting them to cross the blood retinal barrier and spontaneously induce EAU^11^. In those studies, it was unclear whether the IRBP-TCR Tg mice were lymphopenic or whether they developed spontaneous colitis, but evidence of activated IL17-producing cells in the gut was reported. In the present study, we suggest that retinal antigen (HEL)-specific activation of Tg TCR/HEL cells in the inflamed colon may be possible via potential presentation of MHCII-HEL complexes by LP granulocytes (Figs. 4 and 6; Suppl. Table S6; Suppl. Fig. S5). Indeed, in previous work, we have shown that sTg TCR specific T cells require to be activated by presentation of specific antigen in order to become pathogenic^22^. Granulocytes are now recognised as potent antigen presenting cells and selectively activate T effector memory cells^48^ which are the dominant T cell population in the LP gut. How might HEL antigen be presented in situ in the colon? Cell-associated retinal antigen is released to the periphery both constitutively^17,25^ and during inflammation and is likely to traffic to the gut, in its role as a major secondary lymphoid organ^49^. Antigen-specific dTg TCR/HEL T cells which are present in numbers in the dTg LP, might thus be activated by granulocyte-associated HEL. They may also be activated through dual expression of a non-Tg endogenous TCR as has been shown in the Tg TCR/OVA mouse^16,50^. In support of this mechanism, we have previously shown a requirement for endogenous TCR engagement in the development of EAU in the model reported here^17^. Both antigenic cross reactivity and dual TCR expression may be possible, and indeed it is likely, that several colon-related mechanisms for activation of retina-specific pathogenic T cells may be involved. Investigation of which antigen(s) (commensal, cross-reactive, enteric, or ectopic) activate(s) colonic T cells is beyond the scope of the present work and should form the basis of future studies taking into account the requirement for HEL-peptide to convert sTg TCR T cells to a pathogenic role.

With regards to the question of the sequence of events in the gut and the eye, our data support an initial lymphopenia-induced activation of pathogenic T cells in the eye, with release of non-conventional HEL-primed APC (granulocytes) to the periphery potentially inducing activation of antigen-specific effector memory T cells which home to the gut. In the gut, IL17+ HEL-specific T cells induce dysbiosis and eventually colitis. This promotes both antigen-specific and non-antigen-specific dTg T cell activation in the gut. Memory effector T cells are then released to the circulation where they may traffic to the eye and further exacerbate the retinitis. A similar process has been described in a MOG antigen-specific model of EAE^51^.

We propose in our spontaneous EAU model that the profound lymphopenia and reduction in Treg in dTg TCR/HEL mice is sufficient to initiate disease in the eye releasing ocular HEL antigen to activate the extensive pool of colonic HEL-specific T cells, ultimately not only causing colitis but feeding back to drive eye inflammation. Although further research is required to definitively prove the concept of ectopic HEL antigen-presentation in the colon, we show strong potential for this to occur which could have implications for future clinical interventions.

## Methods

### Study design

Transgenic mice were used to investigate in detail the immunological dynamics and severity of colitis in the dTg and sTg TCR genotypes, that co-occurs with spontaneous experimental autoimmune uveitis (EAU) in dTg mice. Age-matched WT (B10.Br) and sTg HEL mice were used as controls. For histological studies, mice were P30 ± 3, for flow cytometry experiments adult mice of different age groups were used (Suppl. Table 1). Median age- and sex distributions were equal across genotype groups (*p* 0.547 and 0.450, respectively).

### Animals

The generation of dTg mice was previously described^17^. All animal research procedures adopted were in agreement with the regulations of the Animal License Act (UK), and were performed under a valid licence and complied with the ARRIVE guidelines. All mice were bred in established breeding colonies and housed in the Medical Research Facility of the University of Aberdeen under a specific-pathogen-free (SPF) controlled environment. Genotypes of the mice were verified by using standard in-house PCR procedures. Littermate male and female mice of different genotypes were used in the experiments as specified, with sTg HEL or WT mice, respectively, serving as control animals. For all respective experiments, adult male and female mice of the same age were used (Suppl. Table S1).

### Clinical evaluation of ocular disease

Mice fundi were imaged using an otoscope-based fibre-optic light device as described previously^52^. Following the LASA guidelines (Laboratory Animal Science Association’s good practice guidelines for administration of substances), mice were anaesthetized with an intraperitoneal injection of a mixture of 40 mg/kg Ketamine hydrochloride “VETALAR” (Fort Dodge Animal Health Ltd., Southampton, UK) and 20 mg/kg Medetomidine “DOMITOR” (Orion Pharma, Espoo, FI) diluted in injectable water. Administration was performed using single-use needles and syringes. Pupils were dilated with 1% (w/v) Tropicamide, and 2.5% (w/v) Phenylephrine hydrochloride “MINIMS” (both from Bausch & Lomb UK Ltd., Kingston-upon-Thames, UK). Carbomer 2 mg/g liquid gel “VISCOTEARS” (Alcon Eyecare UK Ltd., Camberley, UK) was applied to the corneal surface to protect the cornea from drying during imaging. Anaesthesia reversal was performed by subcutaneous injection of 16 mg/kg Atipamezole “ANTISEDAN” (Orion Pharma, Espoo, FI) in injectable water, during which mice were kept in a warm environment and continuously monitored until complete recovery. Severity of disease was graded on the appearance and number of fundus inflammatory lesions using a scoring system modified from^52^ and an atrophy scoring system previously used and published^53^.

### Flow cytometry

Single cell suspensions of cells isolated from P27–P90 retinas, lymph nodes, thymi^54^, and colon LPMC (lamina propria mononucleated cells)^55^ were analysed using flow cytometry. Both eye-draining (submandibular) and colon-draining (mesenteric) lymph nodes (LN) were processed separately. For analysis of eye-infiltrating cells, retinal tissue was manually separated from the choroid under a dissection microscope. Retinas were digested over 40 min at 37 °C in 1 ml PBS containing a final conc. of 10 μg/ml Liberase and 10 μg/ml DNase I (both from Roche, Mannheim, GE). Dissociated cells were washed and re-suspended in PBS containing 2% FBS for staining. Primary antibodies used (from BD Biosciences, Oxford, UK unless stated otherwise) were as follows: *Fc*-receptors were blocked over 10 min (4 °C) using CD16/32 (clone 2.4G2) antibody (1 µl/1 × 10^6^ cells). In all experiments, a fixable live/dead cell stain was used (eFluor 506, Biolegend, London, UK), to discriminate between dead (stained) and live (unstained/low-staining) cells. Staining was performed over 30 min in PBS using a 1/1000 dilution of the dye at 4 °C (500 µl per 1 × 10^6^ cells) in FACS tubes. Stained cells were then washed in 1 ml PBS (300 g, 5 min, 4 °C) and surface stained with directly conjugated monoclonal antibodies in FACS buffer [0.5% (v/v) bovine serum albumin (BSA), and 2 mM EDTA in Ca^2+^/Mg^2+^ free phosphate buffered saline (PBS) (all from Gibco, Fisher Scientific UK Ltd., Loughborough, UK)]. Intracellular staining for transcription factors was completed thereafter over 120 min, following a fixation/permeabilization step using a buffer kit according to instructions (FoxP3/Transcription factor staining buffer set, eBioscience, Hatfield, UK). For characterization of relevant T cell populations present in LPMC, retinas, smLN, mLN and thymi of mice, the panel included CD4-APC-Cy7 (clone GK1.5), CD25-PE (clone PC61), CD73-AF700 (clone Ty11.8; Biolegend, London, UK), FoxP3-APC (clone FJK16S; isotype ctrl. rat IgG2a kappa-APC, eBioscience Invitrogen), Vβ8.1/8.2-BV605 (clone MR5-2) and FR4-PerCp-Cy5.5 (clone 12A5, Biolegend, London, UK). In addition, to detect HEL peptide [46–61] MHC class II complexes in retinas, smLN, mLN, thymi, and LPMC of mice, a separate myeloid cell panel comprised C4H3 (rat mAb to HEL peptide [46–61] and the I-A^k^ MHC class II antigen (secondary staining with APC; clone Poly4054, Biolegend, London, UK), CD11b-APC-Cy7 (clone M1/70), CD11c-AF700 (clone HL3), F4.80-PE (clone BM8, Biolegend, London, UK), Gr-1-BV605 (clone AL-21), and CD3-FITC (clone 17A2, Biolegend, London, UK) was used. The monoclonal rat C4H3 IgG2b antibody is intellectual property of Dr. Ronald Germain, NIAID/NIH; IC Reference # 2007–090. Depending on the type of experiment 2 × 10^5^–1 × 10^6^ total events were acquired on a BD LSR Fortessa flow cytometer (BD Bioscience, Oxford, UK) with respective antibodies titrated accordingly. Generated data were analysed using FlowJo®, LLC for Windows, version 10 (TreeStar Inc., Ashland, Oregon, US). Populations of interest (total, leukocytes, or LPMC) were gated on a linear or logarithmic forward scatter (FSC-A) *vs.* side scatter (SSC-A) dot plot, followed by exclusion of cell aggregates using a forward scatter pulse gate (FSC-H *vs.* FSC-A). All subsequent analyses were based on live cells only, where unstained (live) cells served as the gating control. Gates for individual markers of interest were set based on fluorescence-minus-one (FMO) or isotype-controls respectively, with acceptable background/unspecific staining signals of ≤ 1% of the parent. Gating strategies for lymphoid and myeloid populations are to be found in the supplementary material to this article (Suppl. Figs. S1 and S2). Compensation had been completed successfully prior to each experiment using UltraComp Beads (Invitrogen, UK) and antibodies with respective fluorochromes.

### Histology and assessment of colitis

Mice (aged 30 ± 3 days) were killed and whole colons immediately removed. The tips of the caeca were cut off, after which bowels were flushed with cold PBS using a 10 ml disposable syringe and a gavage needle, to remove all faecal matter. Colons were then placed on a cold surface, kept well-hydrated using cold PBS, and opened longitudinally using scissors and forceps. "Swiss rolls" were generated by tightly rolling up colons manually (lumen facing inwards) from the rectum to the caecum using forceps. 28 G injection needles were used to hold the rolls together during fixation in 3.7% methanol-free buffered formalin (Sigma Aldrich, UK) over 24 h, followed by another 24 h fixation in a 1% preparation. Fixed samples were subsequently dehydrated in a series of ascending concentrations of ethanol (25, 50, 75% over 15 min each) and kept in 100% ethanol until use. For sectioning (8 µm thick), rolls were re-hydrated in 75% ethanol, embedded in paraffin wax (Paraplast, Sigma Aldrich, UK) for standard haematoxylin and eosin (H&E) staining following the SOP protocol from the NHS Grampian pathology department. Images were collected using a Zeiss Axioscan Z1 slide scanner at a 20 × magnification and evaluated for the presence and severity of inflammation using the Zen lite software (both from Carl Zeiss AG, Oberkochen, DE). Post-acquisition the images remained unchanged with reference to contrast and colour. The severity of colon inflammation was graded using an established protocol (with modifications; Suppl. Table S2)^18^. Additionally, colon length (Fig. 1a) was measured using a standard surgical ruler.

### Immunohistochemistry

Following collection and preparation of colons and eyes of dTg and sTg TCR mice (i.e. genotypes with different aspects/degrees of colitis, with and without EAU), organs were embedded in optimal cutting temperature (OCT) medium (Fisher Scientific, UK). Tissues were fully submerged in OCT and quickly frozen using isopentane (Fisher Scientific, UK) over dry ice until completely white and solid. Blocks were kept frozen at −20 °C and wrapped in aluminium foil until cryostat sectioning (8 µm thick, −23 °C). Cut sections were molten onto Polysine® glass slides (Thermo Fisher, UK) and allowed to dry overnight. Slides were stored back-to-back in aluminium foil at −20 °C until use. For immunostaining, defrosted sections were fixed in ice-cold acetone over 15 min (4 °C), followed by 5 min of re-hydration in Tris-buffer (TBS: 90% 1 M saline, 10% dH_2_O, 0.5% pH 6.8 tris in dH_2_O, 0.1% Tween-20), 15 min each of streptavidin and biotin blocking (Vector Laboratories, 2BScientific, UK) respectively. Unspecific sites (IgG, *Fc*-receptors CD16 and CD32) were blocked over 30 min using a 1 × dilution (in Tween-free TBS) of fish gelatin blocking agent (Biotium, CA, US) and 1 µL *Fc-*block (BD Biosciences) per section. Primary antibody (rat-anti-chicken HEL; monoclonal C4H3, see above), ready biotinylated antibodies (against MHC II, CD4, Gr1/Ly6, polyclonal lysozyme/HEL), or respective (biotinylated) isotype controls were kept on over 90 min at RT. Antibody sources were as follows: MHC II, Biotin-rat x mouse I-A/I-E (clone M5/114.15.2); Biotin-rat x mouse Gr1/Ly6C (clone RB6-8C5); Biotin-rat x mouse CD4 (clone RM4-4; all from Biolegend, London, UK); Biotin-rabbit x chicken Lysozyme (Rockland Immunochemicals, 2BScientific, UK). For secondary labelling (for mC4H3), its isotype control (purified rat IgG2b kappa; Biolegend, UK), and as “secondary only” control, affinity-purified, mouse pre-adsorbed rabbit-anti-rat biotinylated IgG (Vector Laboratories, 2BScientific, UK) was used over 60 min. Respective antibody dilutions are provided on the respective figures and in accompanying captions. Intracellular peroxidase activity was quenched using freshly prepared 3% H_2_O_2_ in dH_2_O over 20 min, followed by streptavidin-HRP (horseradish peroxidase; Agilent, Dako, UK) treatment over 30 min (1:350 in Tween-free TBS). For signal development (over 5–8 min), Vector NovaRed (Vector Laboratories, 2BScientific, UK) was used according to manufacturer instructions. For counterstaining, Vector Haematoxilin QS (Vector Laboratories, 2BScientific, UK) was used over 4 min followed by three tap water rinses. Ready stained sections were dehydrated in 75% ethanol (30 s), followed by 100% ethanol (30 s), and two Histoclear clearing steps (2 min each; National Diagnostics, UK), mounted in hard-set non-aqueous mounting media (VectorMount, Vector Laboratories, 2BScientific, UK) and covered with glass cover slips (22 × 50 mm; Menzel Gläser, DE). Imaging was completed using a slide scanner at 20 × magnification (see above).

### Statistical analyses

Statistical analysis was performed using IBM SPSS Statistics 28 (IBM Corp. Released 2021. IBM SPSS Statistics for Windows, Version 28.0. Armonk, NY: IBM Corp; URL: https://www.ibm.com/uk-en/products/spss-statistics). The Kolmogorov–Smirnov test was used, and histograms generated to check data distribution. With one exception (i.e., comparison of Peyer’s patches) non-parametric distribution was assumed for all data and accordingly, data are presented as medians and inter-quartile range (IQR). For analysis of variance and subsequent median comparisons between groups, the Kruskall-Wallis *H*-test (ANOVA on Ranks) and the Wilcoxon-Mann–Whitney *U*-test, respectively, were applied on all data. For the above mentioned normally-distributed Peyer’s patches dataset, T-test statistics were used. To establish relationships between variables, linear bivariate correlations were calculated using the procedure of Spearman's *rho*. To estimate a potential causative relationship of independent variables and the observed variation in their dependent counterparts (clinical outcomes), the model of bivariate stepwise linear regression analysis was used (probability of F: “entry” 0.05, “exclusion” 0.01). Asterisks denote significant *p-*values based upon a 95% level of confidence (*p < 0.05).

### Ethical approval

This study was completed under a valid licence (P4E0DA9C6) granted by the Home Office. Study plans had been approved by the Medical Research Facility of the University of Aberdeen, prior to commencement of experimental animal work.

## Supplementary Information

Supplementary Information.

## Data Availability

The dataset generated during and/or analysed during the current study are available from the corresponding author on reasonable request.
