# Supplementary material for: Colitis in a transgenic mouse model of autoimmune uveitis may be induced by neoantigen presentation in the bowel
Source: Sci Rep. 2023 Jan 23;13:1256. doi: 10.1038/s41598-022-27018-9 (PMC9870966; doi:10.1038/s41598-022-27018-9)
Supplement: Supplementary file 1 — Supplementary Information. [file 41598_2022_27018_MOESM1_ESM.pdf]

Colitis in a transgenic mouse model of autoimmune uveitis may be induced by neoantigen presentation in the bowel

Christine Mölzer[1, 2], Yi-Hsia Liu[1, 3], Elizabeth Muckersie[1], Izabela P. Klaska[1, 4], Richard Cornall[5], Heather M. Wilson[1], Lucia Kuffová[1, 6], John V. Forrester[1, *]

[1]*University of Aberdeen, Institute of Medical Sciences, Foresterhill, Aberdeen, AB25 2ZD, UK.*
[2]*Present address: Medical University of Vienna, Vienna General Hospital, Department of General Surgery, Division of Visceral Surgery, Währinger Gürtel 18-20, 1090 Vienna, AT.*
[3]*Present address: University of Glasgow, Wolfson Wohl Cancer Research Centre, Flow Facility, Switchback Road, Bearsden, Glasgow G61 1BD, UK.*
[4]*Present address: Centre for Gene Therapy and Regenerative Medicine, Guy's Hospital, Great Maze Pond, London, SE1 9RT, UK.*
[5]*Nuffield Department of Medicine, University of Oxford Henry Wellcome Building for Molecular Physiology, Old Road Campus, Headington, Oxford, OX3 7BN, UK.*
[6]*Department of Ophthalmology, Aberdeen Royal Infirmary, NHS Grampian, Aberdeen, UK.*

*Correspondence:
Prof. John V. Forrester; University of Aberdeen, Institute of Medical Sciences, Foresterhill, Aberdeen, AB25 2ZD, UK; Tel. +44 (0)1224 437507; j.forrester@abdn.ac.uk.

Supplementary Figures

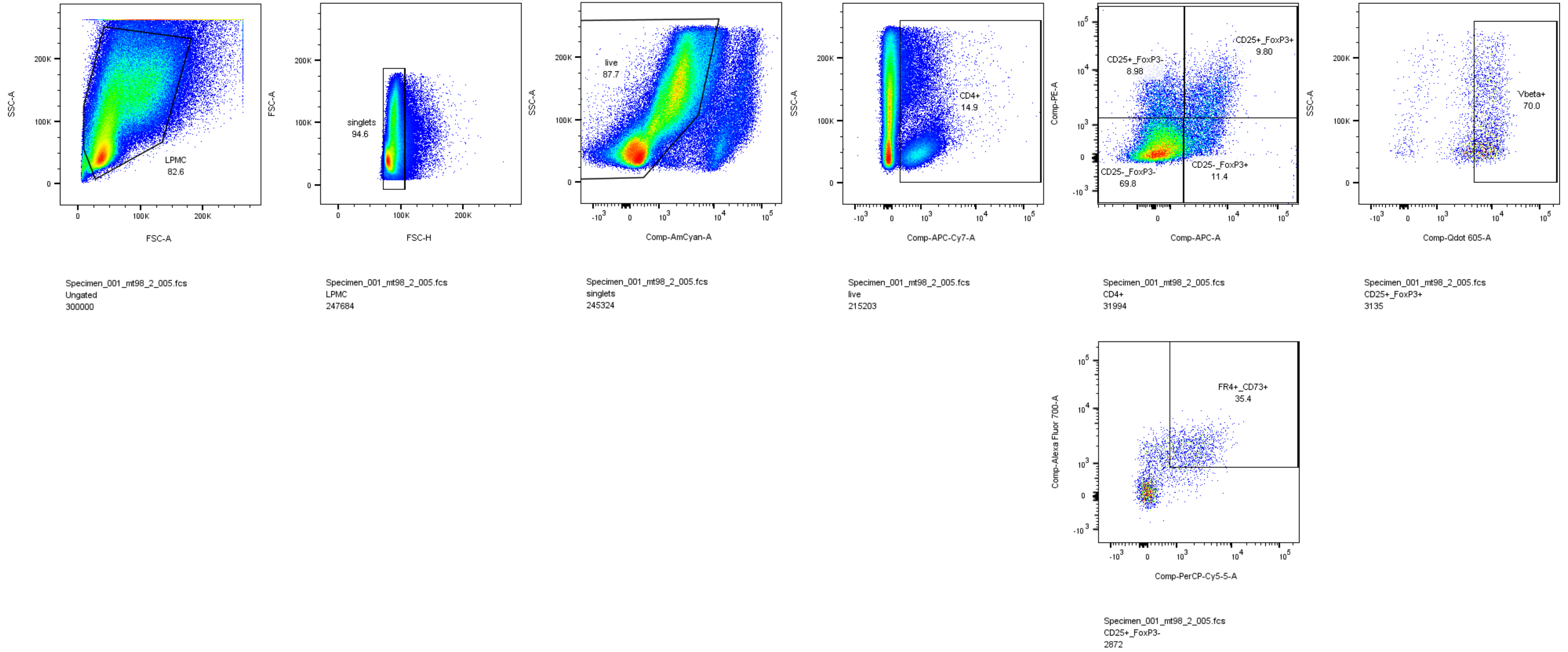

Supplementary Figure S1. ***General T cell gating strategy for LPMC, retina, smLN, and mLN.*** Events had been acquired on a BD LSRII Fortessa cytometer. Data analysis was performed in FlowJo (Treestar Inc.). Populations of interest were **Tconv:** CD4+ CD25+/CD25-; **antigen-specific Tconv:** CD4+ CD25+/- Vbeta8.1/8.2+; **Treg:** CD4+ CD25+ FoxP3+ FR4+/-; **ag-specific Treg:** CD4+ CD25+ FoxP3+ FR4+/- Vbeta8.1/8.2+; **Tan** CD4+ CD25+ FR4+ CD73+. In each experiment (2-3 repetitions), 5-7 mice were used per tissue and genotype group. Abbreviations: LPMC, lamina propria mononucleated cells; smLN, submandibular lymph nodes; mLN, mesenteric lymph nodes.

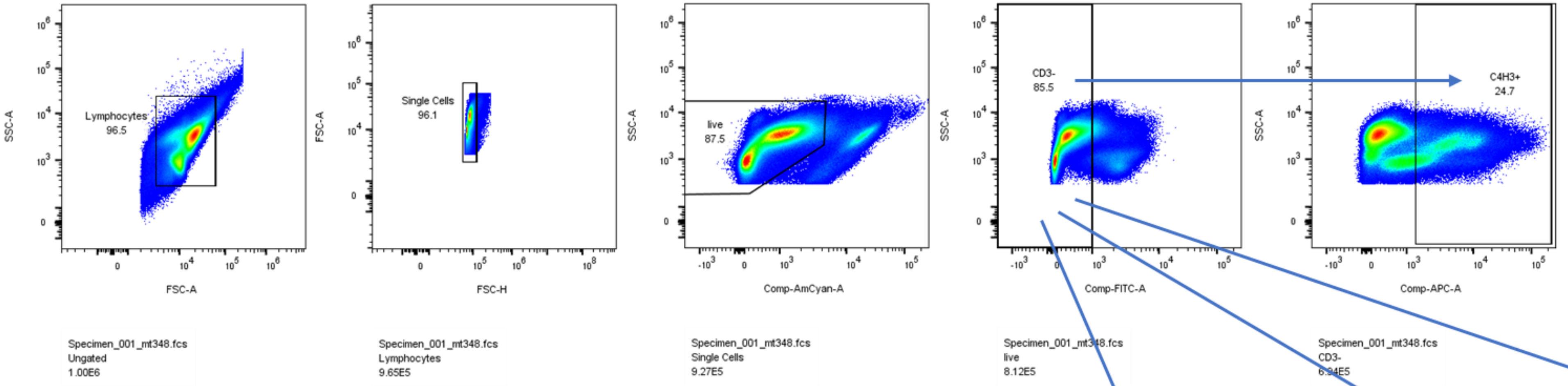

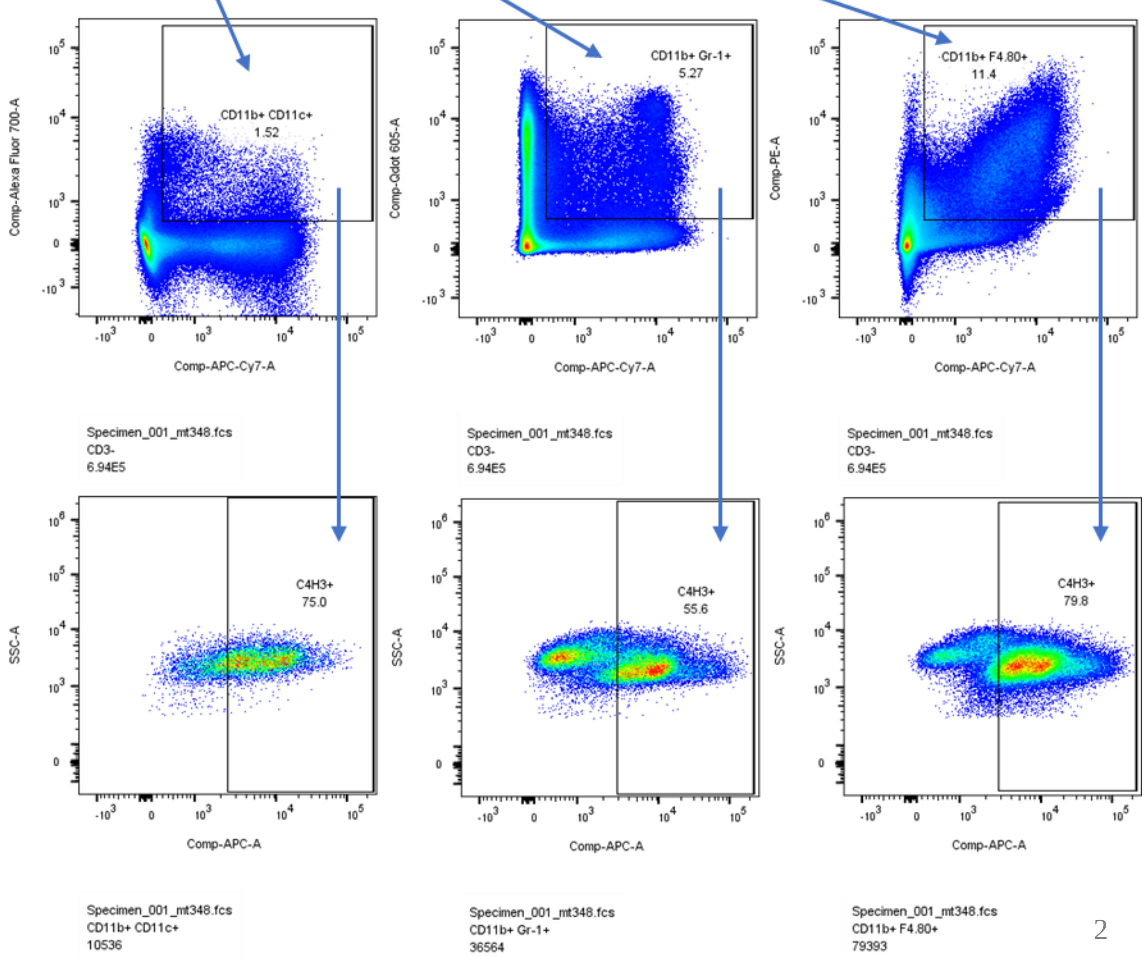

Supplementary Figure S2. ***General myeloid cell gating strategy for LPMC, retina, smLN, and mLN and thymus.*** Events had been acquired on a BD LSRII Fortessa cytometer. Data analysis was performed in FlowJo (Treestar Inc.). Gating of HEL peptide [46-61] I-A^k MHC class II complexes (C4H3) was done in CD3- events as well as in cDC, neutrophils/granulocytes, and glia cells/macrophages. Populations of interest were **conventional DC:** CD3- CD11b+ CD11c+; **neutrophils/granulocytes:** CD3- CD11b+ Gr-1+; **microglia/macrophages:** CD3- CD11b+ F4.80+. In each experiment (2-3 independent repetitions), 5-7 mice were used per tissue and genotype group. Abbreviations: LPMC, lamina propria mononucleated cells; smLN, submandibular lymph nodes; mLN, mesenteric lymph nodes.

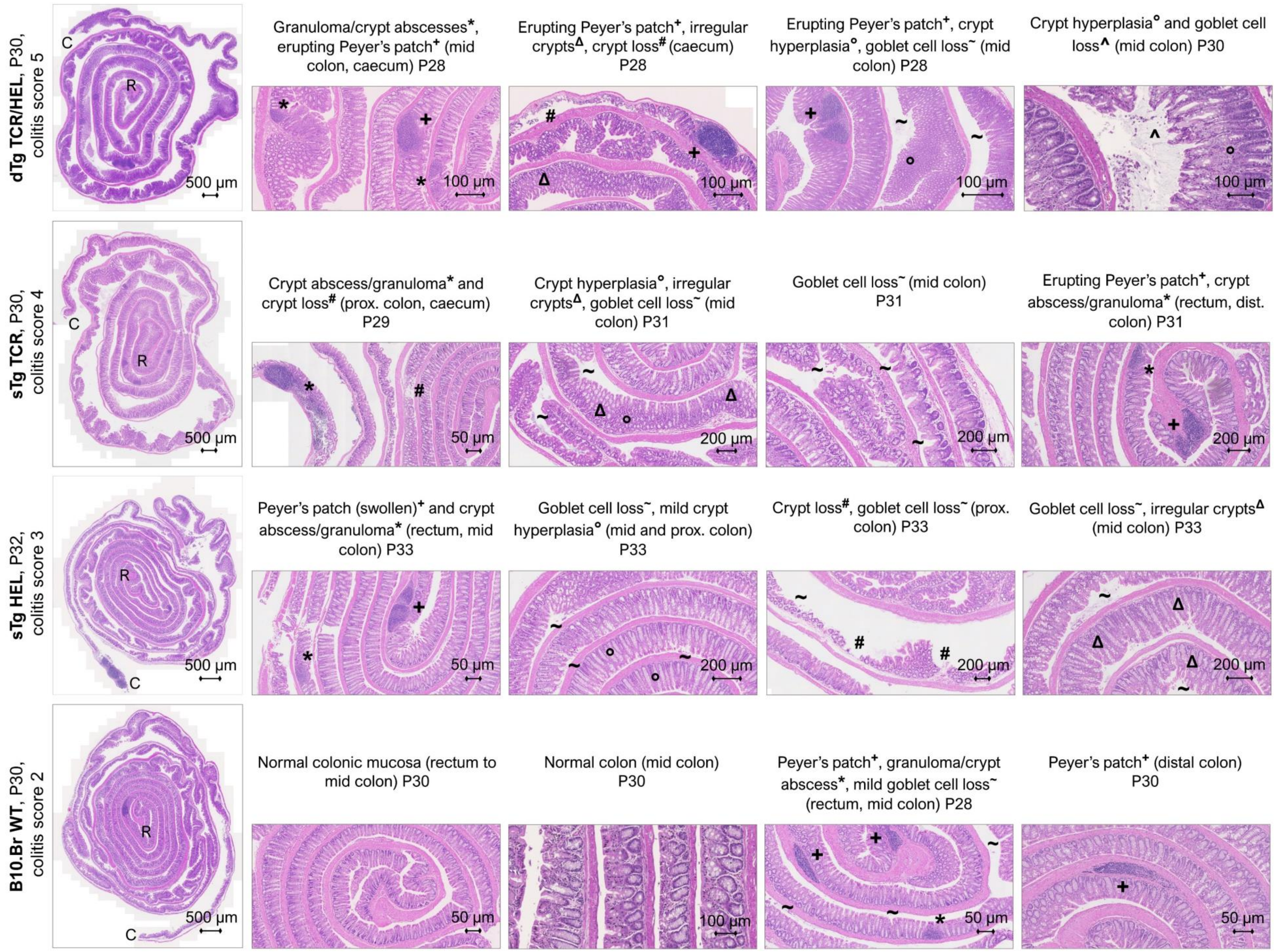

Supplementary Figure S3. *Histology of H&E-stained bowel Swiss roll sections of P30 (± 3 post-partum days) old mice.* For each genotype (n=10-13/group, independently assessed), entire cross sections as well as areas of interest are presented and described. Severity scoring of bowel inflammation was performed based on H&E-stained colon Swiss roll sections (8 µm thick) and Supplementary Table S1.

This chart shows the microscopic morphological appearance of the cardinal features of granulomatous colitis found in the TCR/HEL mouse model of spontaneous EAU. These include granulomas/crypt abscesses, erupting Peyer's patches, crypt hyperplasia/loss/irregularity, and goblet cell loss.

Note abundance of inflammatory signs in dTg TCR/HEL and sTg TCR mice (score 4 – 5, marked to severe). Colons of sTg HEL and WT (B10.Br) mice had normal appearance with only minor inflammation (score 2 – 3; mild to moderate) and served as controls. Abbreviations: R, rectum; C, caecum.

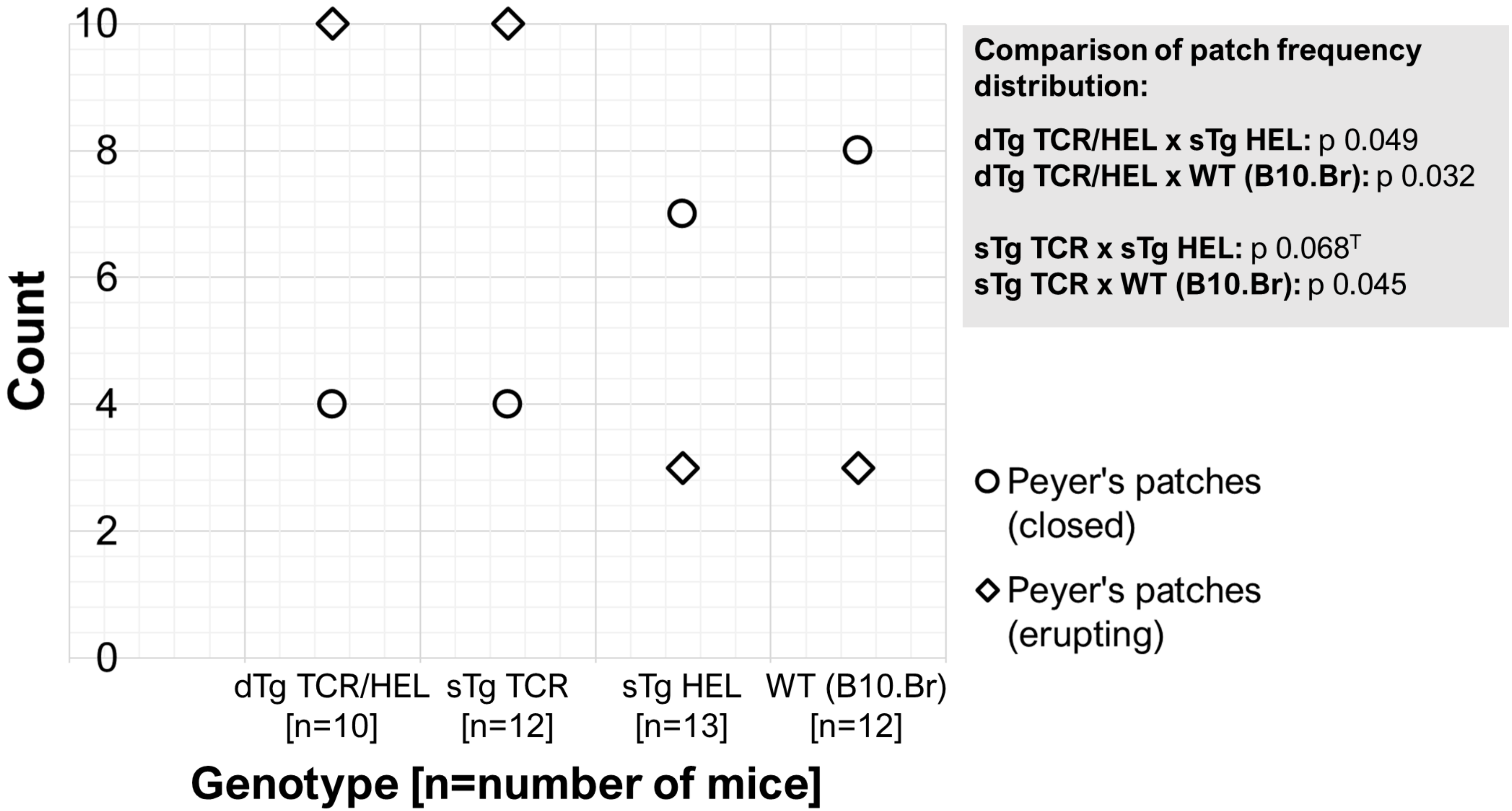

Supplementary Figure S4. ***Quantification of Peyer's patches in H&E-stained colon Swiss roll sections and frequency distribution across genotypes.*** Peyer's patches ("closed" vs. "erupting") were counted in n=10–13 sections per genotype, independently assessed. Mean counts are displayed in the graph and significantly differed between genotype clusters (i.e., cluster 1: dTg and sTg TCR vs. cluster 2: sTg HEL and WT mice); grey box. Higher numbers of erupting along with fewer closed Peyer's patches were found in dTg and sTg TCR mice. The inverse was true for sTg HEL and WT mice. Parametric statistical procedures were used, $p \leq 0.05$; ^{T}denotes a trend ($p \leq 0.1$).

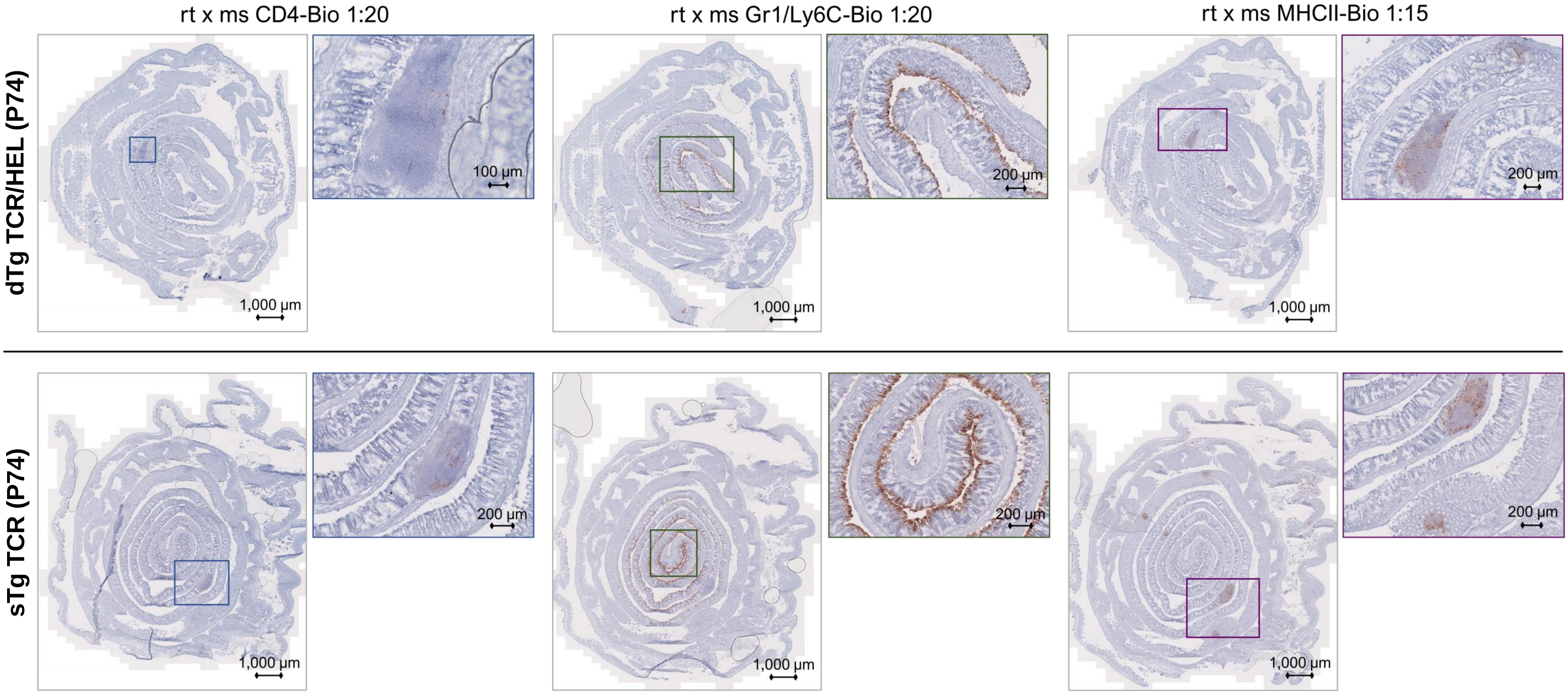

Supplementary Figure S5. ***Immunostaining in mouse colon Swiss roll sections.*** Haematoxylin QS-stained sections of dTg (top panel) and sTg TCR mice (bottom panel) were stained for CD4 T cells, Gr1/Ly6 granulocytes and MHCII-expressing antigen-presenting cells. Note brown positive signal across both genotypes. As expected, CD4 cells were relatively less abundant in lymphopenic dTg sections, granulocyte staining was most pronounced in the distal colon in both genotypes, and MHCII expression was most marked in granulomatous aggregates. Control sections (isotype ctrl: 1:400 rt IgG 2B kappa + 2° 1:5,000 rb x rt IgG-Bio) were free of positive staining (for ease of reading not shown). Per genotype group n=3 sections were independently stained across three replicates.

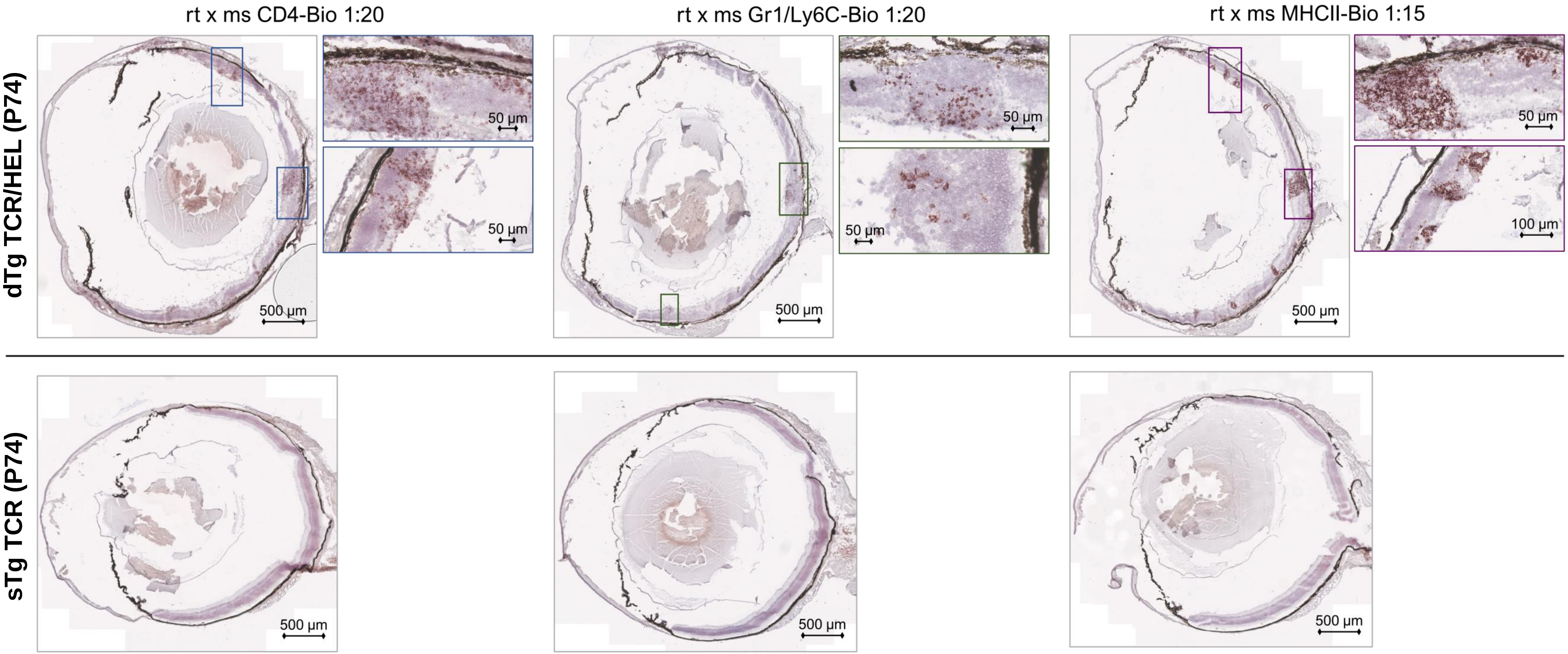

Supplementary Figure S6. ***Immunostaining in mouse eye sections.*** Haematoxylin QS-stained retinal sections of dTg (top panel) and sTg TCR (bottom panel) mice were stained for CD4 T cells, Gr1/Ly6 granulocytes and MHCII-expressing antigen-presenting cells. Note brown positive signal in dTg eyes. As expected, all immune cells of interest had infiltrated inflamed dTg retinas, while uninflamed sTg TCR eyes were completely devoid of them. Control sections (isotype ctrl: 1:400 rt IgG 2B kappa + 2° 1:5,000 rb x rt IgG-Bio) were free of staining (for ease of reading not shown). Per genotype group n=3 sections were independently stained across three replicates.

Dep. Variable	Indep. corr. variables [n]	Excluded confounding variables	Explanatory variable R^2 and p value *dTg TCR/HEL*	Explanatory variable R^2 and p value *sTg TCR*
Colitis	12	Age, mLN immune cells.	Ag-spec. Treg in retina R^2 0.342, p 0.001	Tconv in retina (from circulation) R^2 0.950, p 0.003
Colon length	12	Age, mLN and LP immune cells.	Ag-spec. Treg in retina R^2 0.269, p 0.021	Mice have normal colon length.
Retinitis	8	Retinal immune cells.	Treg in smLN R^2 0.367, p 0.001	Mice have no retinitis.

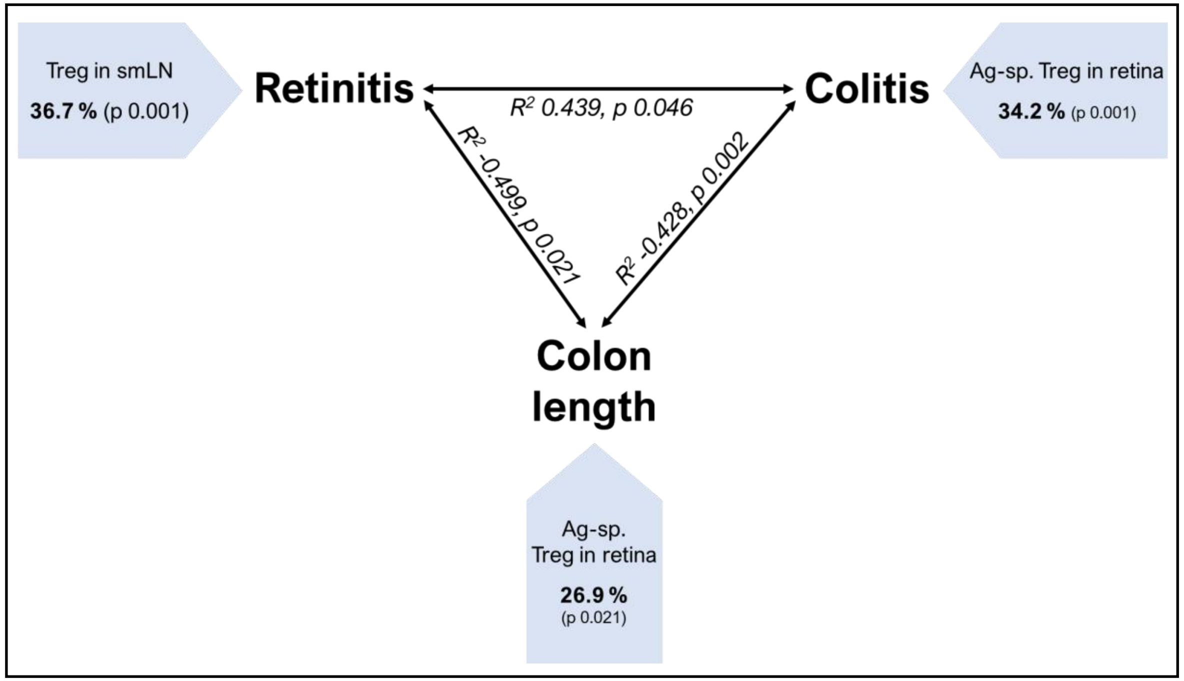

Supplementary Figure S7. ***Model summary.*** The cartoon illustrates the statistical relationship of clinical and immunological parameters of interest. Two-sided arrows (centre) indicate significant bivariate correlations between endpoint variables across all genotypes, while blue boxy arrows highlight independent variables with potential explanatory power (causality) for variation in clinical endpoint markers of interest (dependent variables) in the dTg genotype only. Non-parametric statistical procedures were used, $p \leq 0.05$. Note, median age and sex distribution did not differ across genotype groups (p 0.547 and 0.450, respectively). Abbreviations: mLN, mesenteric lymph node; smLN, submandibular (eye-draining) lymph node; LP, lamina propria.

Supplementary Tables

Genotype	Total n	n [m]	n [f]	Age [d]	Colon length [cm]	Colitis [score 1-5]	Retinitis [score 0-5]	Atrophy [score 0-4]
**dTg TCR/HEL**	34	21	13	36.5 (11.0)	9.3 (1.3)	4.5 (1.0)	2.3 (2.3)	0.0 (0.0)
**sTg TCR**	35	16	19	39.0 (17.0)	9.8 (1.5)	4.0 (1.0)	0.0 (0.0)	0.0 (0.0)
**sTg HEL**	29	17	12	33.0 (14.0)	10.0 (1.1)	3.0 (1.0)	0.0 (0.0)	0.0 (0.0)
**WT (B10.Br)**	36	17	19	34.5 (11.0)	9.5 (2.1)	3.0 (2.0)	0.0 (0.0)	0.0 (0.0)

Supplementary Table S1. ***Description of mice studied.*** The table describes total numbers (n) used, sex distribution (m/f), median age in days (inter-quartile range in brackets) and clinically relevant parameters (colon length, histological colitis, retinitis and retinal atrophy. For histology, colon Swiss roll sections (8 µm thick) were H&E stained and scored according to Supplementary Table S2. Retinitis and retinal atrophy were scored according to a published scoring system, [modified from Xu et al., 2008 [1]] and an atrophy scoring system previously used by us [2]; median scores for both eyes are presented (inter-quartile range in brackets).

Inflammatory cell infiltrate					
**Severity**	**Location/extent**	**Epithelial changes**	**Granuloma/crypt abscess**	**Mucosal architecture**	**Score**
**Minimal**	Mucosa	Minimal hyperplasia	-		**1**
**Mild**	Mucosa ± submucosa	Mild hyperplasia, minimal goblet cell loss ± erosions	max. 1		**2**
**Moderate**	Mucosa + submucosa	Moderate hyperplasia, ± few crypt abscesses/granulomas, moderate goblet cell loss ± erosions	≤ 3		**3**
**Marked**	Mucosa + submucosa	Marked hyperplasia ± several crypt abscesses/granulomas and/or erosions	≤ 7	± irregular crypts or crypt loss ± ulcerations	**4**
**Severe**	Transmural	Marked hyperplasia ± multiple crypt abscesses/granulomas	≥ 8	± irregular crypts or crypt loss ± ulcerations	**5**

Supplementary Table S2. ***Scoring system for histological evaluation of colon inflammation using H&E-stained colon Swiss roll sections.*** Modified after Erben et al [3].

	dTg TCR/HEL [n=10] Genotype 1	sTg TCR 2 [n=12] Genotype 2	sTg HEL 3 [n=13] Genotype 3	WT (B10.Br) [n= 12] Genotype 4
**Peyer's patches Ratio** [closed:erupting]	[1:2.5]	[1:2.5]	[2.3:1]	[2.7:1]
**Peyer's patches Score** $[(c+e)/n]^{+}$	2.4	2.0	1	1.2

Supplementary Table S3. ***Frequency and distribution of Peyer's patches across genotypes.*** Closed (c) and erupting (e) Peyer's patches (Pp) were independently counted in n=10-13 H&E-stained bowel Swiss roll sections per genotype group. Ratios [closed:erupting] and numerical scores were calculated. $^{+}$For every closed Pp a score of 1 was used, for every erupting Pp the value was 2. Non-parametric statistical procedures were used, $p \leq 0.05$.

Spearman *rho* correlations across the entire model:

(Genotype x Pp Score): R^2 -0.380, p 0.008.

(Colon inflammation x Pp Score): R^2 0.676, p 0.001.

	dTg TCR/HEL	sTg TCR	sTg HEL	WT (B10.Br)
LPMC				
Treg [n]	4,856 (7,934)	1,503 (2,194)	797 (2,315)	3,018 (1,038)
Treg [% of CD4]	13.7 (11.4)	3.9 (6.0)^{T}	3.0 (6.3)	7.8 (3.6)
Tconv [n]	12,471 (13,268)	31,183 (21,980)^{T}	19,312 (16,023)	26,758 (7,321)^{T}
Tconv [% of CD4]	40.1 (9.0)	87.4 (12.7)*	76.8 (12.1)*	76.5 (11.5)*
Ag-sp. Treg [n]	2,939 (6,367)	1,414 (1,251)	50 (250)*	431 (211)^{T}
Ag-sp. Treg [% of CD4]	8.2 (12.4)	3.4 (3.5)	0.6 (1.0)*	1.5 (0.5)*
Ag-sp. Tconv [n]	4,735 (6,102)	4,331 (6,144)	1,099 (1,615)	2,196 (993)^{T}
Ag-sp. Tconv [% of CD4]	13.2 (5.6)	12.5 (10.9)	6.5 (4.3)*	6.1 (2.5)*
Tan [n]	101 (116)	46 (151)	459 (463)^{T}	172 (99)*
Tan [% of CD4]	0.4 (0.4)	0.3 (0.4)	1.8 (1.2)*	0.5 (0.3)*
Submandibular LN				
Treg [n]	1,484 (644)	1,764 (1,570)	2,739 (1,306)^{T}	2,420 (1,476)
Treg [% of CD4]	14.4 (5.0)	5.4 (2.9)*	6.1 (2.1)*	6.1 (0.9)*
Tconv [n]	7,019 (4,562)	28,636 (24,593)*	15,283 (33,149)	29,654 (38,674)
Tconv [% of CD4]	69.1 (35.3)	85.1 (8.0)*	42.6 (73.2)*	79.9 (77.3)*
Ag-sp. Treg [n]	1,460 (651)	1,380 (1,168)	525 (658)^{T}	469 (157)*
Ag-sp. Treg [% of CD4]	14.1 (5.1)	4.3 (3.7)*	1.1 (1.8)*	1.0 (0.2)*
Ag-sp. Tconv [n]	5,445 (3,685)	18,660 (21,662)*	3,166 (10,337)*	4,544 (6,177)*
Ag-sp. Tconv [% of CD4]	53.6 (28.7)	76.4 (62.8)	6.7 (27.6)*	12.4 (12.2)*
Mesenteric LN				
Treg [n]	1,020 (297)	1,353 (688)*	2,548 (935)*	2,594 (1315)*
Treg [% of CD4]	10.4 (1.9)	4.7 (1.3)*	5.1 (1.1)*	4.8 (1.3)*
Tconv [n]	6,931 (2,932)	25,342 (15,457)*	42,425 (10,516)*	40,302 (14,327)*
Tconv [% of CD4]	65.8 (18.5)	89.9 (3.0)*	85.1 (4.5)*	85.0 (5.5)*
Ag-sp. Treg [n]	995 (360)	1176 (662)	460 (283)^{T}	423 (204)*
Ag-sp. Treg [% of CD4]	9.5 (2.3)	4.3 (3.2)*	0.9 (1.1)*	0.9 (0.2)*
Ag-sp. Tconv [n]	5,306 (3,021)	20,719 (14,316)*	7,529 (3,863)*	6,884 (2,320)*
Ag-sp. Tconv [% of CD4]	50.1 (22.5)	77.4 (61.5)	14.1 (16.6)*	15.0 (2.1)*
Retina				
Treg [n]	1,446 (1,264)	2.0 (7.0)*	2.0 (5.0)*	3.0 (12.0)*
Treg [% of CD4]	17.4 (6.7)	0.6 (9.6)*	0.3 (1.3)*	1.0 (7.0)*
Tconv [n]	6,142 (3,785)	226 (266)*	230 (296)*	172 (290)*
Tconv [% of CD4]	71.2 (14.5)	90.4 (3.2)*	89.9 (8.7)*	89.0 (10.0)*
Ag-sp. Treg [n]	1,419 (1,264)	1.0 (1.0)*	0.0 (1.0)*	0.0 (2.0)*
Ag-sp. Treg [% of CD4]	17.0 (6.9)	0.3 (1.0)*	0.0 (0.1)*	0.0 (0.8)*
Ag-sp. Tconv [n]	3,781 (4,210)	24 (29)*	14 (13)*	16 (36)*
Ag-sp. Tconv [% of CD4]	44.0 (24.6)	13.8 (13.5)*	5.8 (9.7)*	7.8 (10.5)*

Supplementary Table S4. ***CD4 T cell populations across different tissues and genotypes.*** Numbers [n] provided are median absolute cell counts standardised as per $2x10^5$ recorded events, or percentages of all CD4+ cells [% of CD4] recorded by flow cytometry. Inter-quartile ranges are provided in brackets. Non-parametric statistical procedures were used; *indicates significant difference to dTg mice (p≤0.05), ^{T}denotes a trend difference to dTg mice (p≤0.1). Per genotype group n=5-7 mice were used across 2-3 independent experiments.

Tconv: CD4+ CD25+/CD25-; **antigen-specific Tconv:** CD4+ CD25+/- Vbeta8.1/8.2+; **Treg:** CD4+ CD25+ FoxP3+ FR4+/-; **ag-specific Treg:** CD4+ CD25+ FoxP3+ FR4+/- Vbeta8.1/8.2+; **Tan** CD4+ CD25+ FR4+ CD73+.

	dTg TCR/HEL	sTg TCR	sTg HEL	WT (B10.Br)
**LPMC**				
Tconv/Treg ratio [based on n]	2.6 (7.7)	22.5 (205)*	24.2 (18.8)^{T}	10.1 (4.4)*
Ag-sp. Tconv/Treg ratio [based on n]	1.6 (4.5)	4.9 (48.1)	15.2 (33.2)^{T}	6.5 (3.4)^{T}
**Submandibular LN**				
Tconv/Treg ratio [based on n]	4.4 (2.9)	13.6 (12.0)*	5.3 (10.2)*	11.6 (11.2)^{T}
Ag-sp. Tconv/Treg ratio [based on n]	3.5 (2.3)	15.6 (7.5)*	5.0 (9.3)*	10.8 (11.7)*
**Mesenteric LN**				
Tconv/Treg ratio [based on n]	6.8 (1.3)	19.5 (5.0)*	16.9 (3.6)*	17.4 (4.7)*
Ag-sp. Tconv/Treg ratio [based on n]	5.3 (1.5)	18.4 (2.0)*	16.2 (2.8)*	16.9 (2.9)*
**Retina**				
Tconv/Treg ratio [based on n]	3.7 (1.8)	162.0 (217.0)*	130.4 (156.0)*	95.3 (341.6)*
Ag-sp. Tconv/Treg ratio [based on n]	2.8 (0.6)	24.0 (29.0)*	14.0 (17.8)*	11.0 (27.5)*

Supplementary Table S5. ***Ratios of (antigen-specific) [Tconv/Treg] populations across different tissues and genotypes.*** Values provided are quotients of [Tconv/Treg] and [Ag-sp. Tconv/Treg], based on median absolute cell counts [n] standardised as per $2x10^5$ events recorded by flow cytometry. Inter-quartile ranges are provided in brackets. Non-parametric statistical procedures were used; *indicates significant difference to dTg mice ($p\leq0.05$), ^{T}denotes a trend difference to dTg mice ($p\leq0.1$). Per genotype group n=5-7 mice were used across 2-3 independent experiments.

Tconv: CD4+ CD25+/CD25-; **antigen-specific Tconv:** CD4+ CD25+/- Vbeta8.1/8.2+; **Treg:** CD4+ CD25+ FoxP3+ FR4+/-; **ag-specific Treg:** CD4+ CD25+ FoxP3+ FR4+/- Vbeta8.1/8.2+.

	dTg TCR/HEL	sTg TCR	sTg HEL	WT (B10.Br)
**Retina**				
DC	6,299 (5,273)	42 (46)*	91 (20)*	53 (51)*
C4H3+ DC	5,224 (6,183)	36 (27)*	74 (11)*	44 (37)*
Macrophages	37,350 (27,690)	1,386 (180)*	2,378 (1,390)*	1,511 (871)*
C4H3+ Macrophages	34,152 (24,459)	1,221 (141)*	2,226 (1,534)*	1,296 (654)*
Granulocytes	18,880 (23,433)	171 (187)*	181 (231)*	116 (277)*
C4H3+ Granulocytes	16,178 (20,410)	136 (161)*	155 (174)*	67 (190)*
**Submandibular LN**				
DC	4,101 (1,710)	2,673 (950)	2,375 (1,817)	2,013 (1,061)
C4H3+ DC	1,996 (2,409)	1,955 (682)	1,985 (1,480)	1,401 (1,067)
Macrophages	31,462 (33,512)	23,711 (16491)	17,729 (15,108)*	15,830 (10,538)*
C4H3+ Macrophages	25,527 (18,368)	19,199 (12,263)	17,288 (14,453)^T	14,462 (10,642)*
Granulocytes	19,155 (9,865)	6,286 (12,141)^T	7,935 (8,675)*	8,003 (1,840)*
C4H3+ Granulocytes	12,358 (7,519)	5,821 (10,126)*	7,369 (7,862)*	7,211 (2,259)*
**Mesenteric LN**				
DC	5,098 (4,650)	5,834 (5,196)	2,783 (5,031)	338 (451)*
C4H3+ DC	1,327 (3,888)	5,639 (4,705)	2,083 (4,508)	243 (355)*
Macrophages	40,169 (24,051)	30,235 (22,990)	9,685 (18,413)*	1,609 (13,079)*
C4H3+ Macrophages	32,195 (24,515)	28,836 (20,561)	8,839 (17,537)*	1,596 (12,318)*
Granulocytes	22,583 (9,435)	6,378 (11,782)*	4,590 (4,566)*	1,240 (5,504)*
C4H3+ Granulocytes	18,562 (13,290)*	6,165 (11,398)*	4,011 (4,419)*	1,166 (4,567)*
**LPMC**				
DC	10,298 (8,678)	10,294 (9,259)	10,157 (15,532)	13,695 (10,053)
C4H3+ DC	9,151 (7,616)	8,166 (7,381)	9,794 (12,487)	10,018 (9,864)
Macrophages	10,0017 (56,208)	79,160 (38,891)	93,615 (32,770)	95,082 (29,911)
C4H3+ Macrophages	99,673 (60,417)	72,724 (30,859)	84,934 (40,708)	79,722 (32,274)
Granulocytes	47,577 (28,625)	38,845 (16,832)	36,018 (13,939)	37,013 (21,883)^T
C4H3+ Granulocytes	47,243 (31,306)	35,499 (15,148)	31,421 (4,044)	23,195 (8,421)*

Supplementary Table S6. ***Presence of CD3- myeloid cells and corresponding HEL-MHC II complexes across different tissues and genotypes.*** Numbers (n) provided are median absolute cell counts standardised as per 1x10^6 events recorded by flow cytometry. Inter-quartile ranges are provided in brackets. Antigen-presenting cells found in non-inflamed retinas of sTg TCR, sTg HEL and WT mice are circulatory contaminating cells. C4H3 positivity found in genotypes incompetent of HEL expression is non-specific background staining. Non-parametric statistical procedures were used; *indicates significant difference to dTg mice ($p \leq 0.05$), ^{T}denotes a trend difference to dTg mice ($p \leq 0.1$). Per genotype group n=5-7 mice were used across 2-3 independent experiments.

cDC: CD3- CD11b+ CD11c+; **granulocytes/neutrophils:** CD3- CD11b+ Gr-1+; **macrophages/microglia:** CD3- CD11b+ F4.80+.

REFERENCES

1. Xu H, Koch P, Chen M, Lau A, Reid DM, Forrester JV. A clinical grading system for retinal inflammation in the chronic model of experimental autoimmune uveoretinitis using digital fundus images. *Experimental Eye Research* 2008; **87**(4)**:** 319-326.

2. Liu YH, Corbett C, Klaska IP, Makinen K, Nickerson JM, Cornall RJ *et al.* Partial retinal photoreceptor loss in a transgenic mouse model associated with reduced levels of interphotoreceptor retinol binding protein (IRBP, RBP3). *Exp Eye Res* 2018; **172:** 54-65.

3. Erben U, Loddenkemper C, Doerfel K, Spieckermann S, Haller D, Heimesaat MM *et al.* A guide to histomorphological evaluation of intestinal inflammation in mouse models. *Int J Clin Exp Pathol* 2014; **7**(8)**:** 4557-4576.
